# Genotype-Informed Characterization of Mild Renal Hypouricemia

**DOI:** 10.1016/j.ekir.2026.106711

**Published:** 2026-07-22

**Authors:** Noa Carrera, Adela Urisarri, Pedro Fortes-González, Eloísa Sánchez-Cazorla, Yolanda González Piñeiro, Ana Barcia de la Iglesia, Jorge Amigo, Cándido Díaz, Pablo Pedrosa, María García-Murias, Miguel García González

**Affiliations:** 1 Group of Genetics and Developmental Biology of Renal Diseases, Health Research Institute of Santiago de Compostela (IDIS), Santiago de Compostela, A Coruña, Spain; 2 RICORS2040 (Kidney Disease), Carlos III Health Institute, Santiago de Compostela, A Coruña, Spain; 3 Pediatric Nephrology Service, University Hospital of Santiago de Compostela, Santiago de Compostela, A Coruña, Spain; 4 Pediatric Nephrology Service, Hospital Comarcal do Salnés, Vilagarcía de Arousa, Pontevedra, Spain; 5 Galician Public Foundation for Genomic Medicine, Santiago de Compostela, A Coruña, Spain; 6 Nephrology Service, University Hospital of Santiago de Compostela, Santiago de Compostela, A Coruña, Spain

**Keywords:** diagnostic thresholds, mild hypouricemia, renal hypouricemia, *SLC22A12*, *SLC2A9*, uric acid

## Abstract

**Introduction:**

Renal hypouricemia is caused by pathogenic variants in *SLC22A12* and *SLC2A9*. Current diagnostic thresholds based on serum uric acid (UA) (SUA < 2 mg/dl) and fractional excretion of UA (FEUA > 10%) may overlook individuals with monoallelic variants and mild hypouricemia, whose clinical implications remain incompletely defined. This study evaluated genotype-phenotype correlations across individuals with 0, 1, or 2 pathogenic alleles in a referral-based genetic testing cohort from Galicia (Western Europe).

**Methods:**

We analyzed probands referred for suspected renal hypouricemia, their relatives, and additional carriers not previously suspected of the condition. Genetic, biochemical, and clinical data were integrated to characterize SUA and FEUA distributions by genotype and to identify overlooked cases.

**Results:**

Among 21 probands, 15 carried pathogenic or likely pathogenic variants (71% diagnostic yield), including 3 variants not previously linked to renal hypouricemia. Monoallelic carriers frequently showed mild hypouricemia (SUA: 2–3.3 mg/dl), whereas biallelic carriers had SUA < 2 mg/dl. SUA and FEUA showed an allele-dose pattern, although FEUA availability was limited. Additional carriers identified outside renal hypouricemia suspicion had compatible biochemical profiles when data were available, suggesting underrecognition in clinical practice.

**Conclusion:**

In this referral-based cohort, monoallelic pathogenic or likely pathogenic variants in *SLC22A12* and *SLC2A9* were frequently associated with mild hypouricemia, supporting FEUA assessment and follow-up when low SUA is persistent or clinically suggestive. Current diagnostic thresholds may miss some of these individuals, supporting genotype-informed refinement of serum urate criteria to improve detection and monitoring.

## Graphical abstract

**Figure:**
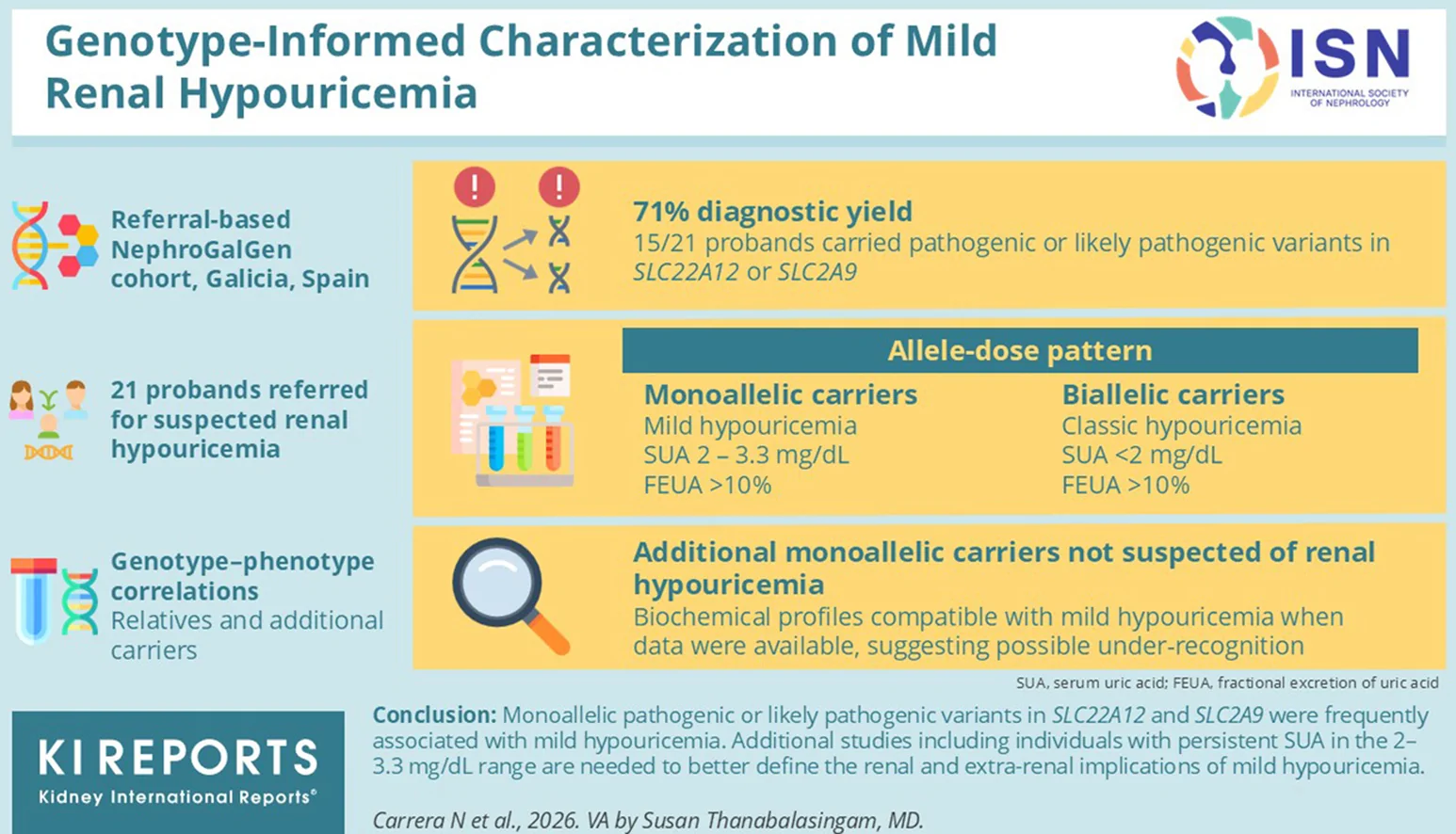

UA, the end product of purine metabolism, acts as an antioxidant in plasma and a prooxidant intracellularly.^1^^,^^2^ Its levels are regulated through production, reabsorption, and excretion to balance physiological roles and prevent harm.^3^ In the kidney, approximately 90% of filtered UA is reabsorbed in the proximal tubule through URAT1 and GLUT9.^4^ FEUA helps maintain SUA within a physiological range, which varies according to age, sex, and other factors.^2^

Defective tubular reabsorption increases FEUA and lowers SUA, causing renal hypouricemia,^5^ a proximal tubular disorder included among rare inherited renal tubulopathies. Hereditary renal tubulopathies are monogenic disorders caused by defects in tubular transport mechanisms,^6^ and renal hypouricemia is listed among proximal tubule disorders involving *SLC22A12* and *SLC2A9.* Renal hypouricemia is mainly because of pathogenic variants in *SLC22A12*^7^ and *SLC2A9*,^5^ which encode URAT1 and GLUT9, respectively. Although classically described as a recessive condition, dominant or codominant inheritance patterns with milder phenotypes have also been reported.^5^^,^^8^

In nephrology practice, clinical attention is often focused on the opposite end of the urate spectrum—hyperuricemia, gout, and the debated links with chronic kidney disease^9^^,^^10^—whereas low SUA values frequently receive less attention.

Low SUA values are often overlooked in routine laboratory testing, and FEUA is rarely measured, so a substantial proportion of cases likely remain undetected. This underrecognition is compounded by the fact that most affected individuals are asymptomatic, particularly during childhood. Reported complications include recurrent nephrolithiasis and exercise- or dehydration-triggered acute kidney injury (AKI), with AKI described in approximately 10% of reported cases.^11^ Detecting renal hypouricemia during AKI can be challenging; however, because SUA may normalize transiently during the episode, masking the underlying disorder.^12^

Additional but less frequent complications or associations reported in individuals with low SUA include hematuria, hypercalciuria, cardiovascular events (SUA: 0.01–4.0 mg/dl),^13^ increased mortality in hemodialysis patients with SUA < 5.0 mg/dl^14^ and chronic kidney disease progression, particularly in women with SUA ≤ 3.5 mg/dl.^15^

Renal hypouricemia is commonly defined by SUA < 2 mg/dl and FEUA > 10%; however, these thresholds remain debated. They reflect the very low SUA characteristic of biallelic cases (often ≤1 mg/dl), whereas growing evidence indicates that monoallelic pathogenic variants can impair UA reabsorption and produce mild hypouricemia, typically in the 2 to 3 mg/dl range.^16^ These values may fall within laboratory reference ranges and are therefore often not investigated, although exercise-induced AKI and other renal hypouricemia-related complications have been reported in individuals with SUA values extending into the <3 to 3.5 mg/dl range.^17^^,^^18^ Consequently, mildly hypouricemic individuals are frequently underrepresented in clinical series, limiting understanding of the full phenotypic spectrum and the clinical implications of monoallelic variants.

Here, we investigated the genetic and clinical features of renal hypouricemia in a referral-based cohort from Galicia, a region with relative historical isolation and a distinctive genetic background.^19^^,^^20^ We focused on individuals with mild hypouricemia, explored overlooked cases (including monoallelic or biallelic carriers with previously unrecognized low SUA), and evaluated genotype-phenotype correlations across pathogenic or likely pathogenic allele counts to inform refinement of SUA-based diagnostic criteria.

## Methods

### Study Participants and Recruitment

This study used the NephroGalGen cohort, a referral-based genetic testing cohort comprising approximately 5400 individuals referred between 2013 and 2025 to either NefroCHUS or the Fundación Pública Galega de Medicina Xenómica for renal and nonrenal indications. NefroCHUS serves as a regional reference center for renal genetic diagnostics, whereas Fundación Pública Galega de Medicina Xenómica provides testing for a broader range of inherited conditions. Both laboratories receive samples from the 12 public hospitals across Galicia (northwest Spain; population: ∼ 2.7 million) within a universal public health care system that provides equitable access to genetic testing when clinically indicated. This framework provides an opportunity to identify renal-related pathogenic variants present in the Galician population, whose demographic history has contributed to a distinctive genetic background.^19^^,^^20^

**Figure 1:**
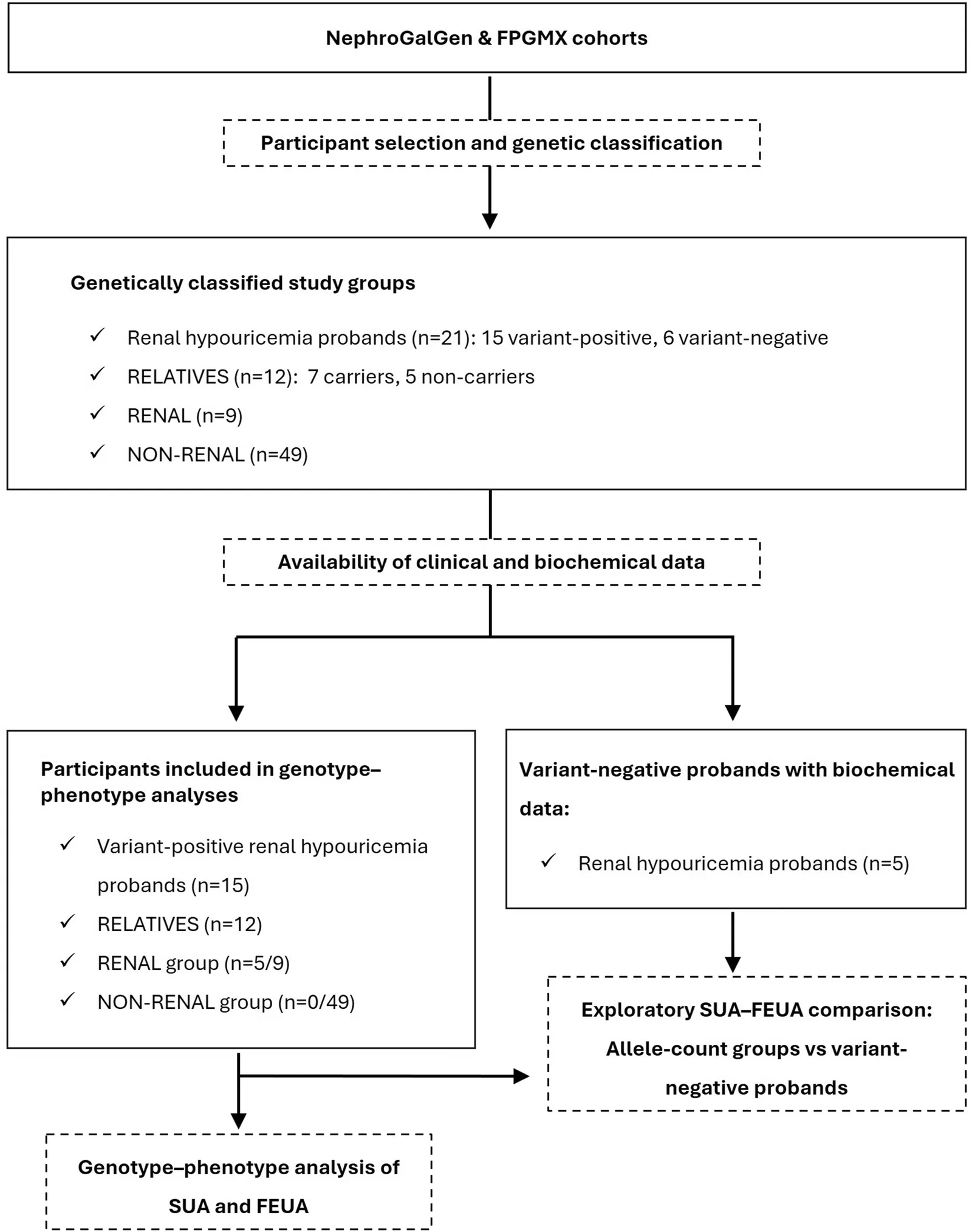
Study workflow and group composition for genotype-phenotype analyses in renal hypouricemia. Individuals referred for suspected renal hypouricemia were selected from the NephroGalGen cohort (*n* = 21). Genetic testing identified pathogenic or likely pathogenic variants in *SLC22A12* or *SLC2A9* in 15 probands, whereas 6 were classified as variant negative. Additional carriers of candidate disease-causing variants were identified through targeted screening of relatives of renal hypouricemia probands (RELATIVES), individuals tested for kidney diseases other than suspected renal hypouricemia (RENAL), and individuals referred for nonrenal genetic conditions whose sequencing included *SLC22A12* and *SLC2A9* (NONRENAL). Clinical and biochemical data were collected when available. The final genotype-phenotype dataset included 15 genetically confirmed renal hypouricemia probands and 5 variant-negative probands, 12 relatives (7 carriers and 5 noncarriers), and 5 RENAL carriers with interpretable biochemical data. For 1 proband and 1 relative, only qualitative biochemical information was available (“low serum uric acid, increased fractional excretion of uric acid”); these individuals supported clinical–genetic interpretation but were excluded from quantitative analyses. This dataset was used to assess clinical–genetic correlations, the relationship between pathogenic or likely pathogenic allele count and urate traits, and the exploratory positioning of variant-negative probands. FEUA, fractional excretion of uric acid; FPGMX, Fundación Pública Galega de Medicina Xenómica; SUA, serum uric acid.

From this cohort, we selected all probands referred for suspected renal hypouricemia, regardless of whether their SUA and FEUA values met classical diagnostic thresholds (SUA < 2 mg/dl, FEUA > 10%). These individuals constituted the suspected renal hypouricemia proband group (Figure 1). Relatives of these probands were invited to participate for cosegregation analyses, constituting the Relatives group.

To assess genotype-phenotype correlations and identify overlooked carriers, we screened the remaining cohort (cases genetically tested for indications other than suspected renal hypouricemia) to identify additional carriers of candidate disease-causing variants, defined here as variants identified in probands and considered candidate explanations for their phenotype. The following 2 sets of carriers were evaluated: (i) individuals from the NephroGalGen renal cohort (Renal group) and (ii) individuals from the Fundación Pública Galega de Medicina Xenómica cohort referred for nonrenal genetic conditions, whose sequencing included *SLC22A12* and *SLC2A9* (Nonrenal group). All participants provided written informed consent. The study was approved by the relevant institutional ethics committees.

### Clinical Characterization

For each participant, we collected clinical information, including age, sex, biochemical data (SUA, FEUA, and plasma and urine creatinine), family history, and disease-related complications (Table 1 and Supplementary Table S1). Sex was recorded as male or female according to the information available in the patients’ medical records. When available, values were recorded at 2 time points: onset (first documented hypouricemia) and follow-up (the most recent available measurement). Because laboratory reference intervals may vary across participating hospitals or laboratories, analyses were based on absolute SUA and FEUA values (i.e., not normalized to local reference ranges), and no laboratory-specific urate thresholds were used for inclusion.

**Table 1:** Clinical and genetic characteristics of renal hypouricemia probands, relatives, and renal carriers

Group	Subject ID	Relationship	Sex	Debut age, yr	SUA (mg/dl)	FEUA (%)	Genetic summary
Renal hypouricemia	F01.S1	PROBAND	F	31.41	0.8	70	*SLC22A12* p.T467M HOM
Renal hypouricemia	F02.S1	PROBAND	F	13.41	1.9	12.4	Variant-negative
Renal hypouricemia	F03.S1	PROBAND	F	34.66	2	22.5	Variant-negative
Renal hypouricemia	F04.S1	PROBAND	M	2.75	2	13.62	Variant-negative
Renal hypouricemia	F05.S1	PROBAND	F	6	1.3	22.25	*SLC22A12* p.T467M HOM
Relatives	F05.S2	Mother	F	19.6	4.25	NA	*SLC22A12* p.T467M HET
Relatives	F05.S3	Father	M	11.75	5.2	NA	*SLC22A12* p.T467M HET
Relatives	F05.S4	Brother	M	2.08	3.475	NA	*SLC22A12* p.T467M HET
Relatives	F05.S5	Grandmother	F	39.41	4.55	NA	Noncarrier
Renal hypouricemia	F06.S1	PROBAND	F	3.33	2.05	9.7	*SLC22A12* p.L144P HET
Relatives	F06.S2	Mother	F	24.16	3.3	NA	Noncarrier
Relatives	F06.S3	Twin	F	3.33	2.45	13.435	*SLC22A12* p.L144P HET
Renal hypouricemia	F08.S1	PROBAND	F	4	NA	NA	*SLC22A12* p.S282L HET
Relatives	F08.S2	Mother	F	NA	NA	NA	*SLC22A12* p.S282L HET
Renal hypouricemia	F09.S1	PROBAND	M	13.75	2.3	11.8	*SLC22A12* p.A476D HET
Renal hypouricemia	F10.S1	PROBAND	M	3.16	3.45	19.25	Variant-negative
Renal hypouricemia	F11.S1	PROBAND	F	3.58	2.6	13.175	*SLC22A12* p.P268S HET
Relatives	F11.S2	Mother	F	NA	3.3	14.4	*SLC22A12* p.P268S HET
Relatives	F11.S3	Father	M	NA	5.1	11.8	Noncarrier
Relatives	F11.S4	Sister	F	NA	2.2	14.4	*SLC22A12* p.P268S HET
Relatives	F11.S5	Grandmother	F	NA	4.3	9.56	Noncarrier
Relatives	F11.S6	Aunt	F	NA	4.5	7.2	Noncarrier
Renal hypouricemia	F12.S1	PROBAND	M	10.25	1.6	15.45	*SLC2A9* p.R171H HET
Renal hypouricemia	F14.S1	PROBAND	F	5	1.9	21.9	*SLC22A12* p.R434C HET
Renal Hypouricemia	F15.S1	PROBAND	F	51.08	3.25^a^	10.65^a^	*SLC2A9* p.T125M HET
Renal hypouricemia	F16.S1	PROBAND	F	16.16	3	13.425	*SLC22A12* p.T467M HET
Renal hypouricemia	F17.S1	PROBAND	M	3.08	0.8	41.55	*SLC22A12* p.V138L HOM
Renal hypouricemia	F18.S1	PROBAND	F	59.33	2.7^a^	NA	*SLC22A12* p.V138L HET
Renal hypouricemia	F19.S1^b^	PROBAND	F	11.33	3.85	NA	*SLC22A12* p.Y275X HET
Renal hypouricemia	F20.S1	PROBAND	F	NA	NA	NA	Variant-negative
Renal hypouricemia	F22.S1	PROBAND	F	12.41	2.2	10.3	Variant-negative
Renal hypouricemia	F24.S1^b^	PROBAND	F	45.16	2.85	NA	*SLC2A9* p.T125M HET
Renal hypouricemia	F26.S1	PROBAND	M	8	1.8	22.9	*SLC2A9* p.T125M HET
Renal	F27.S1	PROBAND	F	22	2.5	NA	SLC22A12 p.P268S HET
Renal	F28.S1	PROBAND	F	26	1.9	17	*SLC22A12* p.S282L HET
Renal	F29.S1	PROBAND	F	22	2.75	NA	*SLC22A12* p.R434C HET
Renal	F30.S1	PROBAND	F	22	2.95	NA	*SLC22A12* p.R434C HET
Renal	F31.S1	PROBAND	M	53	2.7	NA	*SLC22A12* p.L144P HET

FEUA, fractional excretion of uric acid; HET, heterozygous; HOM, homozygous; NA, no data available; SUA, serum uric acid; variant-negative, no pathogenic or likely pathogenic variant detected.

Debut age refers to the age at first available laboratory test showing hypouricemia, or to the first available biochemical evaluation for relatives or noncarriers; hypouricemia may have been present before its first documented detection. SUA and FEUA values refer to the first analysis in which hypouricemia was detected and/or the most recent available analysis, as detailed in Supplementary Table S1. The “Genetic summary” column includes the gene, protein change and zygosity; all variants are referred to transcript NM_144585.4 for *SLC22A12* or transcript NM_020041.3 for *SLC2A9*. Underlined variants have not been previously linked to hypouricemia. *SLC22A12* p.Y275X is probably a novel variant.

a Values obtained while the subject was receiving medication that can significantly affect SUA levels; these values may not accurately reflect baseline levels and should be interpreted with caution.

b IDs that indicate patients carrying other genetic variants likely to affect SUA levels (Supplementary Table S1); therefore, observed uric acid levels may reflect antagonistic genetic effects; therefore, phenotypic interpretation in these individuals is indeterminate and should be made with caution.

Mild hypouricemia was defined as SUA between 2 and approximately 3.3 mg/dl with FEUA > 10%, whereas classic hypouricemia was defined as SUA < 2 mg/dl with FEUA > 10%. The upper bound (∼ 3.3 mg/dl) was used as a pragmatic, data-informed threshold based on the distribution observed in our cohort; at approximately 3.5 mg/dl, values showed overlap between carriers and noncarriers, supporting the use of this range as a trigger for further evaluation rather than a strict diagnostic cutoff. Accordingly, we framed SUA in this interval as prompting FEUA assessment and consideration of genetic testing when clinically appropriate. Medications known to influence SUA (e.g., thiazides) and other potential modifiers, including coexisting genetic variants, were documented.

### Molecular Analyses

Genomic DNA was extracted from whole blood using standard protocols (GenEX Genomic Kit, GeneAll; QIAamp DNA Blood Mini Kit, Qiagen). DNA quantity was assessed using Qubit fluorometry.

All participants underwent next-generation sequencing using targeted panels or whole-exome sequencing, all including *SLC22A12* and *SLC2A9*. Assay details and additional urate-handling genes sequenced are shown in Supplementary Table S1. Single-nucleotide variants and small insertions or deletions were identified and copy number variants were inferred from read-depth data. Carrier testing in relatives was performed by Sanger sequencing.

### Characterization and Classification of Variants

Each candidate variant identified in the probands referred for suspected renal hypouricemia was evaluated, with particular attention to variants not previously linked to the condition. Functional impact was assessed using *in silico* prediction tools (REVEL, PolyPhen-2, CADD, PhyloP), alongside evidence from the literature, available functional data, and population allele frequencies. Cosegregation and genotype-phenotype data were analyzed in probands, relatives, and additional Renal/Nonrenal carriers with available clinical information.

Variant classification and reclassification followed the American College of Medical Genetics and Genomics/Association for Molecular Pathology (ACMG/AMP) guidelines, incorporating ClinGen Sequence Variant Interpretation recommendations and a quantitative framework for combining evidence^21^, ^22^, ^23^ (Supplementary Methods). Only pathogenic or likely pathogenic variants were considered candidate disease-causing variants for downstream analyses. ACMG/AMP–based classification and key supporting evidence are summarized in Table 2 and Supplementary Table S2; the ACMG/AMP criteria applied to the 3 newly associated variants are summarized in Table 3. All variants were independently assessed by 2 geneticists.

**Table 2:** Characterization of candidate pathogenic or likely pathogenic variants identified in renal hypouricemia probands

DNA change	Protein change	VarS	Frk	MAF	Previously linked to renal hypouricemia	Segregation	Other affected carriers	Authors’ classification
*SLC2A9*								
c.512G>A	p.R171H	VUS	VUS	6,8E-06	Y	Y	NA	LP
c.374C>T	p.T125M	VUS	LP	2,4E-05	Y	Y	NA	P
*SLC22A12*								
c.412G>C	p.V138L	LP	VUS	1,0E-04	Y	NA	NA	LP
c.431T>C	p.L144P	VUS	VUS	2,7E-04	Y	Y	Y	LP
c.802C>T	p.P268S	VUS	VUS	8,5E-04	N	Y	Y	LP
c.825C>G	p.Y275X	LP	LP	0	N	NA	NA	P
c.845C>T	p.S282L	VUS	VUS	1,6E-05	N	Y	Y	LP
c.1300C>T	p.R434C	VUS	VUS	1,8E-04	Y	Y	Y	P
c.1400C>T	p.T467M	B	B	1,6E-04	Y	Y	NA	P
c.1427C>A	p.A476D	VUS	VUS	1,0E-04	Y	NA	NA	LP

ACMG/AMP, American College of Medical Genetics and Genomics/Association for Molecular Pathology; B, benign; FEUA, fractional excretion of uric acid; LP, likely pathogenic; N, no; P, pathogenic; SUA, serum uric acid; VUS, variant of uncertain significance; Y, yes.

This table summarizes the 10 distinct variants identified in probands, including DNA and protein changes according to HGVS nomenclature. VarS and Frk indicate the default classifications returned by VarSome and Franklin, respectively. MAF denotes minor allele frequency in the non-Finnish European population in gnomAD v4.1.0 (GRCh38). “Previously linked to renal hypouricemia” indicates whether the variant has been previously reported in association with renal hypouricemia. “Segregation” indicates whether available segregation data support co-segregation with the biochemical phenotype, based on our families and/or previously published pedigrees. “Other affected carriers” indicates whether additional carriers identified in Relatives or Renal screening had available biochemical/clinical data compatible with hypouricemia. “Authors’ classification” indicates the final ACMG/AMP-based classification assigned by the authors after integrating all available evidence. Expanded variant-level information is provided in Supplementary Methods, Supplementary Results and Supplementary Table S2. For *SLC22A12* p.T467M, several heterozygous relatives showed SUA or FEUA values within reference ranges, consistent with incomplete penetrance and/or variable expressivity in monoallelic carriers.

**Table 3:** ACMG/AMP evidence supporting the classification of 3 *SLC22A12* variants newly associated with renal hypouricemia

Variant (HGVS protein; gene)	gnomAD v4.1 PopMax	Key evidence from this study	ACMG/AMP criteria applied^a^	Final classification (authors)
p.Tyr275Ter (*SLC22A12*)	Absent	Identified in 1 proband; predicted truncating/loss of function variant, consistent with an established disease mechanism in *SLC22A12.*	PVS1_VeryStrong, PM2_Moderate	Pathogenic
p.Ser282Leu (*SLC22A12*)	9.0 × 10^-5^	Proband and limited familial segregation consistent with altered urate handling; additional carriers identified, including 1 carrier meeting classic biochemical criteria (SUA < 2 mg/dl with elevated FEUA); residue conserved and supportive computational evidence.	PM2_Moderate, PP1_Supporting, PP3_Supporting, PP4_Supporting, PS4_Supporting	Likely pathogenic
p.Pro268Ser (*SLC22A12*)	1.6 × 10^-5^	Proband and familial segregation consistent with altered urate handling, with carriers showing lower SUA/higher FEUA than noncarriers; additional carriers identified, including 1 carrier with persistently low SUA; residue conserved and computational evidence supportive	PM2_Moderate, PP1_Supporting, PP3_Supporting, PP4_Supporting, PS4_Supporting	Likely pathogenic

ACMG/AMP, American College of Medical Genetics and Genomics/Association for Molecular Pathology; FEUA, fractional excretion of uric acid; LoF, loss of function; PopMax, highest minor allele frequency across gnomAD populations; SUA, serum urate.

a ACMG/AMP criteria: PVS1, predicted null variant in a gene where LoF is a known mechanism of disease; PM2, absent or very low population frequency; PP1, segregation evidence; PP3, computational evidence supporting a deleterious effect; PP4, phenotype specificity; PS4, increased prevalence in affected individuals compared with controls or enrichment in affected individuals. Full variant-level evidence and *in silico* predictors are provided in Supplementary Table S2 and the Supplementary Results.

### Allele-Dose Analyses and Exploratory Assessment of Variant-Negative Probands

To determine whether carrying a single pathogenic variant in *SLC22A12* or *SLC2A9* is sufficient to alter SUA and FEUA, we compared biochemical values across individuals with 0, 1, or 2 pathogenic or likely pathogenic alleles. Plasma and urine creatinine served as negative controls, because these variants were not expected to affect creatinine handling. To minimize bias from urate interpretation modifiers, individuals with major confounders of SUA or FEUA interpretation (e.g., urate-modifying therapy at the time of measurement, advanced chronic kidney disease, or coexisting genetic conditions known to shift SUA) were excluded from allele-count comparisons (Supplementary Table S1 and Supplementary Methods). Given the modest sample size, primary analyses were performed in the full eligible cohort to maximize statistical power; results are displayed with sex-stratified plots to assess consistency of the allele-dose pattern. To address potential confounding by age and sex, we performed complementary sensitivity analyses (including multivariable models for SUA) to assess their contribution relative to pathogenic allele count; analogous exploratory checks were performed for FEUA, acknowledging limited availability (Supplementary Methods).

We first tested whether the affected gene (*SLC22A12* vs. *SLC2A9*) influenced SUA, FEUA, or creatinine values using Mann-Whitney tests with Benjamini–Hochberg correction. Because no significant differences were detected, subsequent analyses focused on allele count only.

Individuals were classified as biallelic (2 pathogenic or likely pathogenic variants in trans), monoallelic, or noncarriers. Noncarrier relatives served as true controls; probands in whom no pathogenic or likely pathogenic variant was identified (variant-negative probands) were excluded from the noncarrier group and from allele-count group comparisons. For comparisons across groups, we used Kruskal-Wallis tests followed by Dunn’s *post hoc* analyses (Supplementary Methods).

To explore whether SUA and FEUA values could provide suggestive information regarding underlying genotype in variant-negative probands, we generated a SUA-FEUA scatter plot, including individuals with known allele counts and overlaid variant-negative probands as an independent exploratory group (no genotype assignment). This exploratory analysis was not intended to establish a molecular diagnosis or to modify the diagnostic yield.

## Results

### Renal Hypouricemia Probands and Genetic Findings

**Figure 2:**
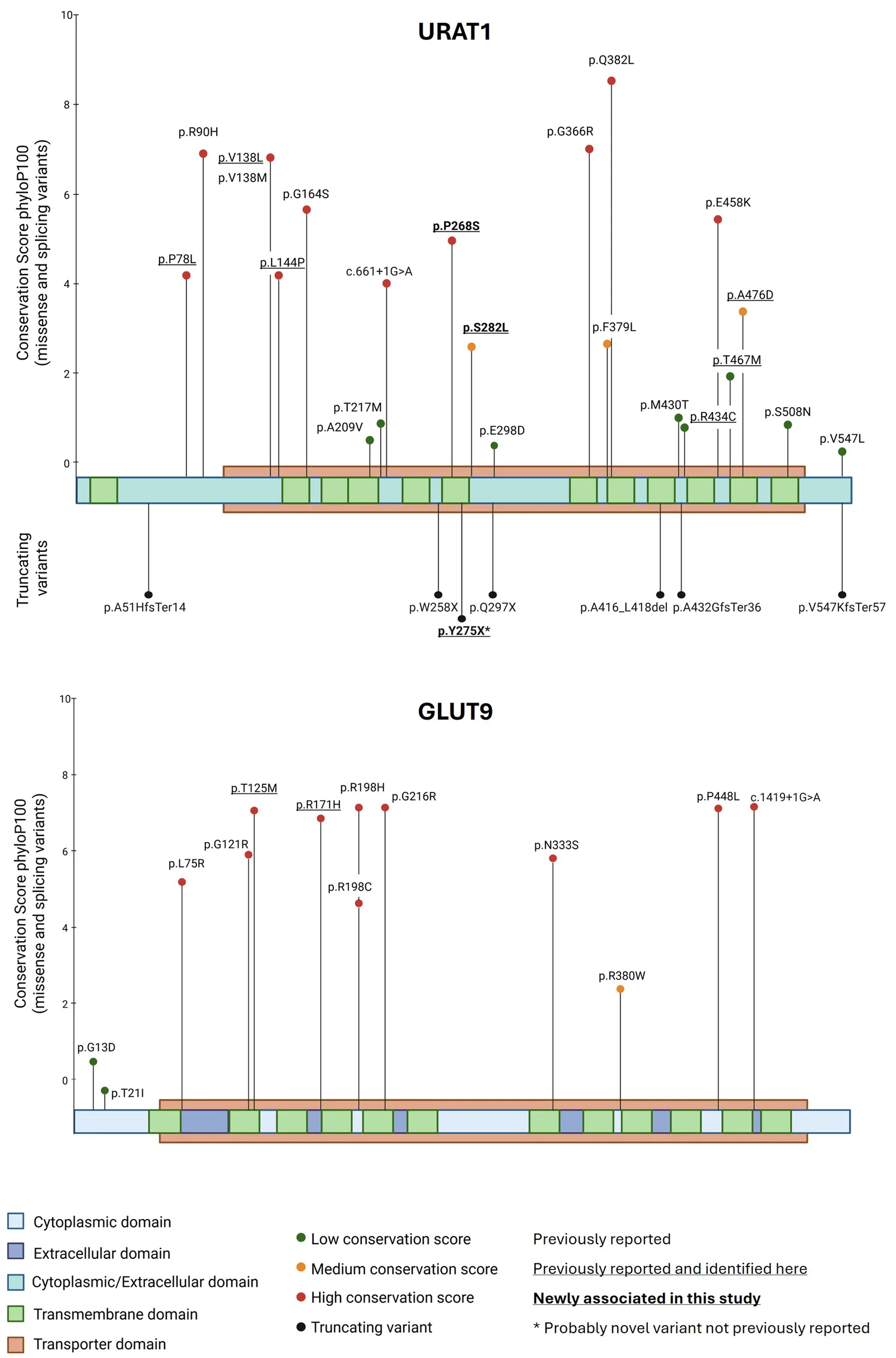
Variants identified in this study and previously reported variants in URAT1 and GLUT9. Schematic representation of the proteins encoded by *SLC22A12* (URAT1) and *SLC2A9* (GLUT9), showing their predicted transmembrane domains and the location of variants identified in this study together with variants previously reported in the literature. Evolutionary conservation is shown as position-specific vertical tracks, with green indicating low conservation, yellow intermediate conservation, and dark red high conservation. Variant labels are formatted according to their relationship with this study and the previous literature: variants previously linked to renal hypouricemia but not identified in this cohort are shown in regular font; variants previously linked to renal hypouricemia and identified in this study are underlined; and variants identified in this study but not previously linked to renal hypouricemia are shown in bold and underlined. The asterisk indicates a likely novel variant not previously reported. All variants are referenced to transcript NM_144585.4 for *SLC22A12* and transcript NM_020041.3 for *SLC2A9*.

We identified 21 probands clinically suspected of renal hypouricemia (Figure 1, Table 1). Candidate disease-causing variants were detected in 15 of 21 probands (71% diagnostic yield): 11 with variants in *SLC22A12* and 4 in *SLC2A9*, comprising 10 distinct variants (8 in *SLC22A12*, 2 in *SLC2A9*) (Table 2). Seven variants had been previously associated with renal hypouricemia, whereas 3 (*SLC22A12*: p.S282L, p.P268S, p.Y275X) are newly associated with hypouricemia in this study. The ACMG/AMP–based classification and supporting evidence are summarized in Table 3 (newly associated variants), and in Supplementary Table S2. The distribution of variants in *SLC22A12* and *SLC2A9*, alongside previously reported renal hypouricemia variants, is shown in Figure 2.

Among the 15 genetically confirmed probands, 3 were biallelic (Table 1): F01.S1 and F05.S1 (homozygous *SLC22A12* p.T467M), and F17.S1 (homozygous *SLC22A12* p.V138L). The remaining 12 were monoallelic carriers.

### Identification of Additional Carriers of Candidate Disease-Causing Variants

Segregation analysis in 12 relatives from 4 families identified 7 monoallelic carriers and 5 noncarriers (Relatives group) (Table 1).

Screening the broader NephroGalGen cohort identified 9 renal carriers (Renal group) and screening the nonrenal Fundación Pública Galega de Medicina Xenómica cohort identified 49 carriers (Nonrenal group), none of whom had been previously suspected of hypouricemia (Figure 1). All 58 were monoallelic carriers of disease-causing variants identified in probands.

Because clinical and biochemical data were unavailable for most additionally identified Renal and Nonrenal carriers, only individuals with interpretable clinical information are reported individually in Table 1 and Supplementary Table S1; the remaining carriers are included only in the carrier-screening summary.

### Clinical Characterization and Genotype–Phenotype Correlations

Clinical data were available for all but 1 proband (F20.S1) (Table 1, Supplementary Table 1). As expected, the 3 biallelic probands showed the classical biochemical profile of renal hypouricemia, with SUA of approximately 1 mg/dl and markedly increased FEUA when available.

Among the 12 monoallelic probands, most exhibited biochemical evidence of impaired urate reabsorption, typically with mild hypouricemia (SUA: ∼ 2–3 mg/dl) and elevated FEUA when measured (Table 1 and Supplementary Table S1). A subset of monoallelic probands (F15.S1, F18.S1, F19.S1, F24.S1) showed atypical SUA or FEUA patterns in the context of major urate-interpretation modifiers (e.g., urate-modifying therapy at the time of measurement, or coexisting genetic conditions known to shift SUA), and these individuals were not considered in allele-count comparisons (Supplementary Table S1, Methods). In particular, phenotypic interpretation in F19.S1, who carried both an *HNF1B*/17q12 deletion and *SLC22A12* p.Y275X; and in F24.S1, who carried biallelic pathogenic *SLC12A3* variants causing Gitelman syndrome together with *SLC2A9* p.T125M, was considered indeterminate because of these competing genetic backgrounds.

In variant-negative probands, paired SUA and FEUA data were available in 5 individuals, with SUA in the 1.9 to 3.5 mg/dl range and FEUA > 10% when available (Supplementary Table S1).

**Figure 3:**
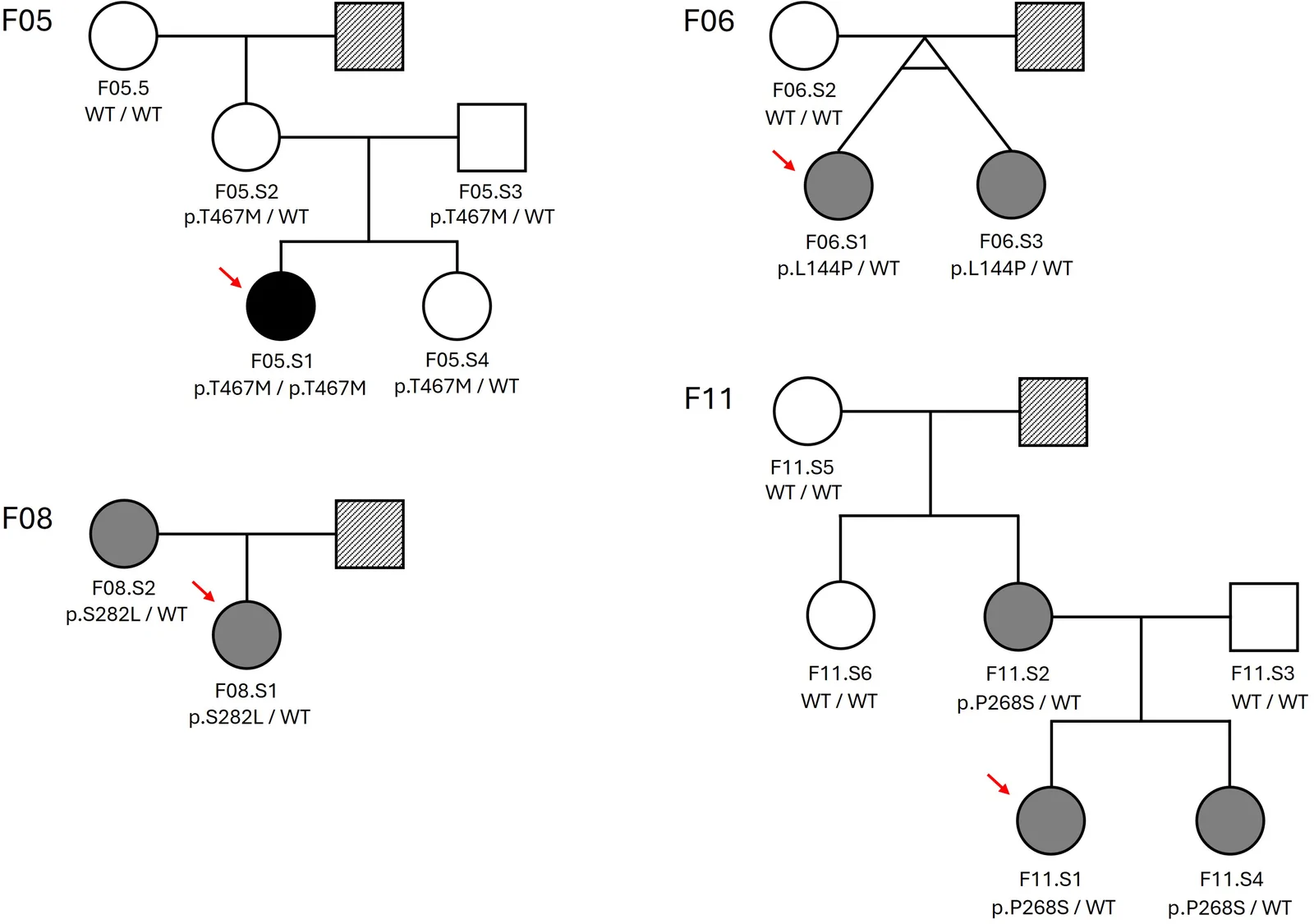
Pedigree and cosegregation analysis of renal hypouricemia-associated variants in 4 families. Pedigrees are shown for the 4 families in which segregation analysis was available. Genotypes are indicated below each individual ID, with WT denoting noncarrier status for the familial variant. Circles represent females and squares represent males. Filled black symbols indicate individuals with classic renal hypouricemia, filled grey symbols indicate individuals with mild renal hypouricemia, and unfilled symbols indicate individuals without renal hypouricemia. Striped symbols indicate individuals for whom biochemical or clinical information was unavailable. Arrows indicate the proband in each family.

Among Relatives, biochemical penetrance in heterozygous carriers was variable: several carriers showed mild hypouricemia, whereas all noncarriers had SUA values within the reference range; notably, heterozygous relatives carrying *SLC22A12 p.T467M* in family F05 showed normal SUA or FEUA, consistent with incomplete penetrance or variable expressivity in heterozygous carriers (Figure 3; Table 1; Supplementary Table S1). In contrast, biallelic p.T467M probands showed the expected marked phenotype. Although automated platforms classify p.T467M as benign, largely because of its relatively high frequency in Roma populations, we retained a pathogenic classification based on published functional evidence demonstrating impaired urate uptake, URAT1 mislocalization, and retention within the endoplasmic reticulum, together with its well-established association with renal hypouricemia, particularly in homozygous or compound heterozygous individuals. Thus, population-frequency evidence was considered in the classification but did not outweigh the combined functional, clinical, and disease-association evidence.

Within the RENAL carrier screening group (*n* = 9), biochemical data were available for 5 individuals, and all of them (all monoallelic) showed SUA values in the mild or classic hypouricemia range (Table 1; Supplementary Table S1). No biochemical or clinical data were available for the remaining Renal carriers or for Nonrenal carriers; therefore, these individuals were not included in Table 1 or Supplementary Table S1.

Outcome data were limited. AKI was documented in 1 variant-negative proband (F02.S1; SUA: 1.8–2 mg/dl; FEUA = 12.4%) following strenuous physical exertion. Nephrolithiasis was recorded in 2 monoallelic probands: F15.S1 had SUA values in the mild hypouricemia range, whereas F18.S1 had discordant SUA measurements and lacked FEUA, limiting biochemical classification. Both cases were excluded from allele-count comparisons because of major urate-interpretation modifiers (urate-modifying therapy in F15.S1) and limited interpretability (discordant SUA and missing FEUA, with concurrent medication in F18.S1).

Detailed variant-level narratives, including genotype-phenotype observations and additional supporting literature, are provided in the Supplementary Results and Supplementary References.

### Phenotypes of Variants Newly Associated With Renal Hypouricemia

We report 3 *SLC22A12* variants newly associated with renal hypouricemia in this study (p.Y275X, p.S282L, and p.P268S). The ACMG/AMP criteria and key supporting evidence are summarized in Table 3, with extended variant-level descriptions provided in the Supplementary Results.

#### p.Y275X

This ultrarare truncating variant (absent from gnomAD v4.1.0) is consistent with a known loss-of-function mechanism in *SLC22A12*, as previously shown for other pathogenic URAT1-disrupting variants.^7^^,^^24^^,^^25^ It was identified in F19.S1, who also carried a pathogenic 17q12 deletion involving *HNF1B*, a condition associated with higher serum urate,^26^ and likely attenuating the expected hypouricemic phenotype. Given this major confounder for SUA interpretation, the case was excluded from allele-count comparisons (Supplementary Table S1, Supplementary Methods).

#### p.S282L

Detected in F08.S1 (a child with pseudohypoaldosteronism, hypouricemia, and hyperuricosuria) (Table 1, Supplementary Table S1). Automated platforms initially classified p.S282L as a variant of uncertain significance, despite *in silico* predictors and residue conservation supporting a deleterious effect (Supplementary Figure S1, Supplementary Table S2). The mother (F08.S2), also a carrier, showed similar mildly low SUA (Figure 3). Screening identified 5 additional carriers; biochemical data were available for only 1 (F28.S1), who had SUA < 2 mg/dl and FEUA of 17%. Taken together, these data supported reclassification of p.S282L as likely pathogenic (Table 3, Supplementary Results).

#### p.P268S

Identified in F11.S1 (SUA: 2.3–2.9 mg/dl, FEUA = 13.35 and 13%) (Table 1, Supplementary Table S1). The variant is ultrarare (2/804,985 individuals in gnomAD), affects a conserved residue (Supplementary Figure S1), and shows deleterious *in silico* scores (Table 3, Supplementary Results); however, automated platforms initially classified it as a variant of uncertain significance. Cosegregation analysis showed clearly lower SUA and higher FEUA in carriers compared with noncarriers (Table 1, Figure 3). Seven additional p.P268S carriers were found in our cohort; clinical data were available only for 1 (F27.S1) with SUA consistently < 3 mg/dl. Overall, these data supported reclassification of p.P268S as likely pathogenic (Table 3, Supplementary Results).

Direct functional validation of p.P268S and p.S282L in heterologous expression systems, such as Xenopus oocytes or HEK293 cells, has not yet been performed. Their classification as likely pathogenic is therefore based on the combined genetic, clinical, segregation, population, and computational evidence, without application of the PS3 functional criterion.

### Allele-Count Effects on SUA and FEUA and Exploratory Assessment of Variant-Negative Probands

We evaluated whether carrying a single pathogenic variant in *SLC22A12* or *SLC2A9* is sufficient to alter SUA and FEUA levels by comparing individuals with 0, 1, or 2 pathogenic or likely pathogenic alleles. Variant-negative probands were excluded from allele-count comparisons and assessed separately as an exploratory overlay (Methods). Individuals with major urate-interpretation modifiers were excluded from allele-count comparisons (Supplementary Table S1).

For allele-count comparisons (boxplots), SUA was available for 26 individuals with known allele count (0/1/2 variants: *n* = 5/18/3) and FEUA for 17 (*n* = 3/11/3). For the SUA-FEUA scatter plot, paired SUA-FEUA values were available for 17 individuals with known allele count, and variant-negative probands with paired data (*n* = 5) were overlaid as an independent exploratory group. Group-level descriptive statistics are reported in Table 4.

**Table 4:** Summary of clinical, biochemical, and genetic findings by study group

Parameter	Groups			
	Renal hypouricemia probands	Relatives carriers	Relatives noncarriers	Renal probands
*n*^1^	21 / 21 (100%)	7 / 7 (100%)	5 / 5 (100%)	5 / 5 (100%)
Sex^1^				
F	15 / 21 (71%)	5 / 7 (71%)	4 / 5 (80%)	4 / 5 (80%)
M	6 / 21 (29%)	2 / 7 (29%)	1 / 5 (20%)	1 / 5 (20%)
Prescribed medication^1^	2 / 21 (9.5%)	0 / 7 (0%)	0 / 5 (0%)	0 / 5 (0%)
Renal complications^1^	3 / 21 (14.3%)	0 / 7 (0%)	0 / 5 (0%)	NA
FH^1^	12 / 60 (63%)	7 / 7 (100%)	5 / 5 (100%)	NA
Biochemical data				
eGFR^2^^,^^3^	115.50 (102.00, 130.75)	147.25 (143.00, 151.50)	NA	NA
Plasma Cr^2^^,^^4^	0.53 (0.41, 0.65)	0.40 (0.29, 0.71)	0.76 (0.71, 0.82)	0.82 (0.80, 0.85)
Urine Cr^2^^,^^4^	107.00 (70.00, 144.00)	77.85 (53.30, 156.00)	73.70 (69.80, 108.70)	61.20 (61.20, 61.20)
SUA^2^^,^^4^	2.05 (1.80, 2.85)	3.39 (2.45, 4.25)	4.50 (4.30, 4.55)	2.70 (2.50, 2.75)
FEUA^2^^,^^4^	14.54 (12.10, 22.38)	14.40 (13.44, 14.40)	9.56 (7.20, 11.80)	17.00 (17.00, 17.00)
Pathogenic variants^1^				
0 alleles	NA	0 / 7 (0%)	5 / 5 (100%)	0 / 5 (0%)
1 allele	12 / 21 (57%)	7 / 7 (100%)	0 / 5 (0%)	5 / 5 (100%)
2 alleles	3 / 21 (14%)	0 / 7 (0%)	0 / 5 (0%)	0 / 5 (0%)
Variant-negative	6 / 21 (29%)	NA	NA	NA

Cr, creatinine; eGFR, estimated glomerular filtration rate; F, female; FEUA, fractional excretion of uric acid; FH, family history, including relatives with hypouricemia, hyperuricosuria, nephrolithiasis, micturition syndrome, or hematuria; M, male; NA, not available or not applicable, as appropriate; SUA, serum uric acid; Variant-negative: no pathogenic or /likely pathogenic variant detected.

Summary statistics are shown for renal hypouricemia probands, RELATIVES carriers, RELATIVES noncarriers, and RENAL carriers with available interpretable clinical/biochemical data.

Categorical variables are reported as *n/N* (%) and continuous variables as median (Q1, Q3). eGFR is expressed as ml/min per 1.73 m^2^; creatinine and SUA as mg/dl; FEUA as %. Prescribed medication refers only to medications that could affect observed SUA levels/interpretation. Renal complications refer to those attributed to renal hypouricemia.

**Figure 4:**
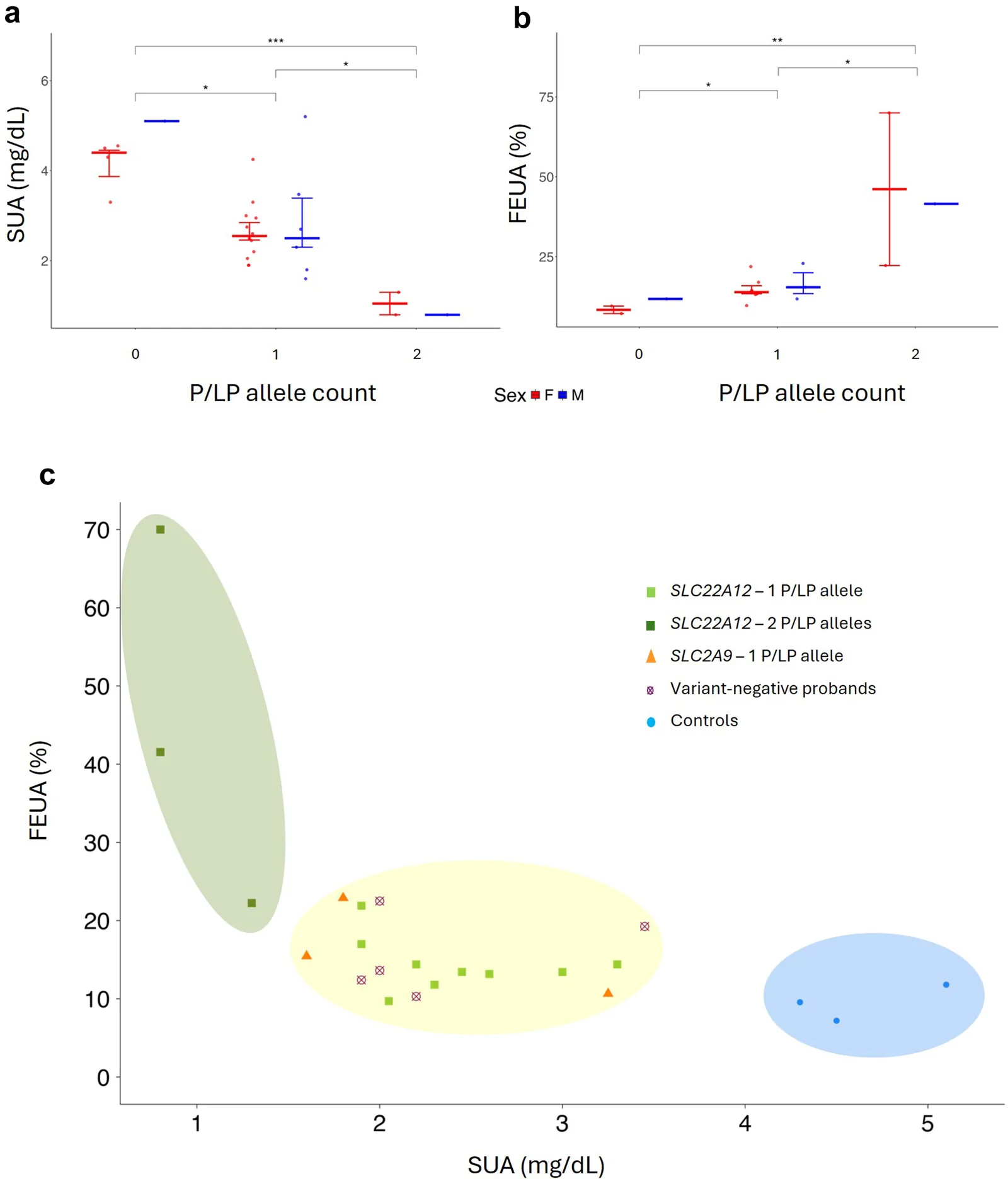
Effect of P or LP allele count on urate traits and exploratory positioning of variant-negative probands. (a and b) Sex-stratified box plots of SUA (mg/dl), and FEUA) (%) according to allele count (0, 1, or 2 P or LP alleles in *SLC22A12* or *SLC2A9)*. Primary comparisons were performed in the full eligible cohort to maximize statistical power; plots are displayed separately by sex to assess consistency of the allele-dose pattern. Individuals with major urate-interpretation modifiers were excluded from allele-count comparisons (Methods and Supplementary Table S1). Available data for group comparisons were as follows: SUA, 0 alleles (*n* = 5), 1 allele (*n* = 18), and 2 alleles (*n* = 3); FEUA, 0 alleles (*n* = 3), 1 allele (*n* = 11), and 2 alleles (*n* = 3). Horizontal bars indicate statistically significant pairwise differences after Kruskal–Wallis and Dunn’s *post hoc* testing: ∗*P* < 0.05, ∗∗*P* < 0.01, ∗∗∗*P* < 0.001. (c) Exploratory scatter plot of SUA versus FEUA, including individuals with known allele count and paired measurements. Variant-negative probands were overlaid as an independent exploratory group and were not included in allele-count hypothesis testing. Points are color-coded as follows: blue, noncarriers (0 alleles); light green, monoallelic *SLC22A12* carriers; dark green, biallelic *SLC22A12* carriers; orange, monoallelic *SLC2A9* carriers; purple, variant-negative probands. Numbers per group were: 0 alleles (*n* = 3), 1 allele (*n* = 11), 2 alleles (*n* = 3), variant-negative probands (*n* = 5). Variant-negative probands fell within the range of monoallelic carriers, consistent with the possibility of an undetected pathogenic variant. FEUA, fractional excretion of uric acid; LP, likely pathogenic; P, pathogenic; SUA, serum uric acid.

Creatinine levels (negative controls) did not differ across allele-count groups and are shown in Supplementary Figure S2. In contrast, SUA differed significantly according to allele count (Kruskal–Wallis *P* = 9.96 × 10^−4^; Figure 4a), with significant pairwise differences between noncarriers and monoallelic carriers (adjusted *P* = 0.0197), noncarriers and biallelic carriers (adjusted *P* = 7.22 × 10^−4^), and monoallelic and biallelic carriers (adjusted *P* = 0.0220). Importantly, the significant difference between monoallelic carriers and noncarriers supports the conclusion that a single pathogenic allele is associated with altered SUA levels. The more extreme values observed in biallelic carriers were consistent with the expected allele-dose pattern; however, pairwise comparisons involving this group should be interpreted cautiously because it comprised only 3 individuals.

FEUA differed by allele count (Kruskal–Wallis *P* = 0.0060; Figure 4b), with pairwise comparisons supporting higher FEUA in monoallelic carriers versus noncarriers (adjusted *P* = 0.0494), biallelic carriers versus noncarriers (adjusted *P* = 0.0042), and biallelic versus monoallelic carriers (adjusted *P* = 0.0494). The significant difference between monoallelic carriers and noncarriers further supports altered urate handling in individuals carrying a single pathogenic allele, although interpretation is limited by incomplete FEUA availability. As for SUA, comparisons involving the 3 biallelic individuals should be interpreted cautiously. The allele-dose pattern was visually consistent in sex-stratified plots; however, the male stratified analysis was considered noninformative because it included only 1 noncarrier and 1 biallelic individual.

To explore whether SUA and FEUA could provide suggestive information regarding underlying genotype in variant-negative probands, we generated a SUA-FEUA scatter plot including individuals with known allele counts and overlaid the 5 variant-negative probands with paired biochemical data as an independent exploratory group (no genotype assignment; Figure 4c). F20.S1 was not included because biochemical data were unavailable. Variant-negative probands fell within the biochemical range observed for monoallelic carriers.

Although alternative genetic causes of hypouricemia were not excluded in all individuals because the genes assessed varied according to the sequencing assay used (Supplementary Table S1), this biochemical similarity is compatible with impaired urate handling of a magnitude comparable to that observed in monoallelic carriers. However, it remains only a suggestive exploratory observation and does not establish an undetected *SLC22A12* or *SLC2A9* variant or any other specific molecular diagnosis. These individuals therefore remained molecularly unresolved, and the diagnostic yield remained 15 of 21 (71%).

## Discussion

Renal hypouricemia is a rare and underdiagnosed disorder caused by impaired UA reabsorption in the proximal tubule, leading to low SUA and increased FEUA. Pathogenic variants in *SLC22A12* and *SLC2A9*, encoding URAT1 and GLUT9, respectively, represent the main genetic causes.^5^^,^^7^^,^^11^ Although many patients remain asymptomatic, renal hypouricemia can present with nephrolithiasis, exercise- or dehydration-induced AKI; and, in some cases, chronic kidney disease.^11^ In contrast to the extensive nephrology literature on hyperuricemia and gout, the clinical implications of low SUA—particularly mild hypouricemia in monoallelic carriers— may receive less attention. Although the phenotype associated with biallelic variants is well-characterized, the clinical impact of monoallelic carriers remains less clearly defined. Our study adds to the growing body of evidence showing that even a single pathogenic variant is sufficient to alter SUA levels,^16^ supporting its clinical relevance. In addition, we expand the variant spectrum of renal hypouricemia by identifying 3 variants newly associated with the disease. Altogether, our findings refine genotype-phenotype correlations, uncover overlooked cases, and broaden the known spectrum of renal hypouricemia-causing variants.

Among 21 probands with clinical suspicion of renal hypouricemia, pathogenic or likely pathogenic variants were identified in 15 (71%), including 3 biallelic and 12 monoallelic cases, involving 10 distinct variants, 3 of which have not been previously associated with renal hypouricemia. This degree of allelic heterogeneity highlights the utility of sequencing-based diagnostics in hypouricemia. Beyond probands, additional monoallelic carriers were detected among relatives and the rest of the cohort (Renal and Nonrenal groups), none of whom had been previously evaluated for hypouricemia. This observation suggests that mildly affected individuals may remain unnoticed in routine clinical practice unless low SUA is specifically investigated or complications prompt further evaluation.

Across all individuals with available genetic and biochemical data, SUA and FEUA patterns confirmed the expected severe phenotype in biallelic cases (SUA close to 1 mg/dl and markedly elevated FEUA) and demonstrated that most monoallelic carriers displayed biochemical abnormalities: 18 of the 24 monoallelic individuals showed altered SUA values (and FEUA, when available). Among them, 14 had mild hypouricemia, with SUA between 2.05 and 3.3 mg/dl and FEUA between 9.7% and 13.4%, including several carriers who had not been clinically monitored for low SUA. The remaining 4 monoallelic carriers presented SUA values slightly < 2 mg/dl together with elevated FEUA, thereby fulfilling classical diagnostic criteria for renal hypouricemia, although their biochemical profiles were less profound than those typically observed in biallelic cases. Together, these findings indicate that monoallelic pathogenic variants frequently produce detectable abnormalities in UA handling, ranging from mild hypouricemia to threshold-defined renal hypouricemia.

The remaining 6 monoallelic carriers displayed atypical biochemical profiles, sometimes reflecting genetic or environmental modifiers. For example, F15.S1, carrying the *SLC2A9* p.T125M variant, initially exhibited low SUA and elevated FEUA but later showed higher SUA coinciding with long-term thiazide therapy, consistent with the urate-retentive effect of thiazides. In F19.S1, the combination of the truncating *SLC22A12* p.Y275X variant with a pathogenic *HNF1B* deletion—known to predispose to hyperuricemia— probably resulted in borderline SUA values due to opposing genetic effects. Similarly, interpretation of SUA in F24.S1 is complicated by the presence of biallelic pathogenic *SLC12A3* variants causing Gitelman syndrome, in addition to the monoallelic *SLC2A9* p.T125M variant. In this case, SUA remained within the mild hypouricemia range, suggesting that the biochemical phenotype may reflect the combined effects of both genetic conditions. F18.S1 (*SLC22A12* p.V138L) showed discordant SUA measurements across 2 time points, and the absence of FEUA data limited interpretation. Finally, the 3 relatives of proband F05.S1, all monoallelic carriers of *SLC22A12* p.T467M, consistently showed SUA and FEUA within the reference range, contrasting with previous reports,^7^ highlighting incomplete penetrance and/or variable expressivity in heterozygous and/or additional modifiers.

These observations highlight that gene-gene interactions, gene-environment factors, and medication effects can substantially influence renal hypouricemia expressivity. Nonetheless, monoallelic carriers showed significantly lower SUA and higher FEUA than noncarriers, supporting the conclusion that a single pathogenic allele is associated with altered urate handling. The more extreme biochemical phenotype observed in biallelic individuals was consistent with the well-established phenotype of biallelic renal hypouricemia and with an allele-dose pattern. However, because only 3 biallelic individuals were included, pairwise comparisons involving this group should be interpreted cautiously and should not be considered precise estimates of the magnitude of the difference between groups. Values persistently below approximately 3 mg/dl—especially when confirmed on repeated measurements—should prompt consideration of FEUA assessment and genetic testing, even in individuals without prior clinical suspicion, where clinically appropriate.

Variant-negative probands with paired SUA-FEUA data fell within the range observed for monoallelic carriers (Figure 4c). Although alternative genetic causes of hypouricemia were not excluded in all cases, the similarity of their biochemical profiles suggests that some may have an underlying defect in urate handling comparable in magnitude to that observed in monoallelic carriers. This interpretation remains exploratory and cannot support assignment to the monoallelic group or establish a specific molecular diagnosis. These individuals therefore remain molecularly unresolved. Future studies using broader genomic approaches are required to investigate other urate-handling genes and noncoding variants that may escape conventional panel or exome analyses, as has been demonstrated in other inherited kidney disorders.^27^

Clinical complications classically associated with renal hypouricemia—particularly nephrolithiasis and AKI—are well-described in individuals with SUA < 2 mg/dl. Evidence for complication risk in mildly hypouricemic individuals remains limited because these patients are often excluded from studies. A prior review^28^ noted that approximately 9% of monoallelic carriers developed nephrolithiasis, approximately 9% AKI, and approximately 6.7% hematuria, although the cohorts included in that review were probably enriched for clinically severe cases. In our cohort, AKI occurred in 1 variant-negative proband with SUA < 2 mg/dl (F02.S1). In addition, nephrolithiasis was documented in 2 monoallelic probands; one of them (F15.S1, *SLC2A9* p.T125M) had mild hypouricemia (SUA: 2.8–3.7 mg/dl, FEUA: 9.3%–12%), whereas the second case (F18.S1, *SLC22A12* p.V138L) showed discordant SUA measurements (4.3 and 1.1 mg/dl) and lacked FEUA data, making classification difficult; and both were excluded from allele-count analyses. Outcome data were limited and retrospectively abstracted; therefore, our cohort is not suitable to estimate absolute complication rates or to validate specific SUA cut-offs. Moreover, these nephrolithiasis cases were excluded from allele-count comparisons because of urate-interpretation modifiers and/or limited biochemical classification (Methods and Supplementary Table S1).

Although limited, the observation of nephrolithiasis in a monoallelic carrier with SUA in the mild hypouricemia range is consistent with prior reports and supports vigilance and follow-up in persistently hypouricemic individuals, rather than risk estimation from our dataset.

Variant classification remains challenging. Existing tools frequently misclassify established renal hypouricemia variants as benign or of uncertain significance, likely because of the underdiagnosis of renal hypouricemia and the consequent underestimation of its true prevalence. In addition, monoallelic carriers with mild phenotypes are rarely represented in clinical cohorts, further complicating classification. Improving variant interpretation will require curated databases, systematic integration of family segregation data, and careful reassessment of the literature, with updated thresholds that account for mild hypouricemia. For the newly associated missense variants, p.P268S and p.S282L, the available evidence supports a likely pathogenic classification, but direct functional confirmation of impaired URAT1 expression, localization, or urate transport remains outstanding. Functional studies in established systems such as Xenopus oocytes or HEK293 cells will be important to strengthen their biological interpretation.

This study has several limitations. Most importantly for the allele-count analysis, the biallelic group included only 3 individuals. Although their markedly low SUA and elevated FEUA were consistent with the well-established biochemical phenotype of biallelic renal hypouricemia, the small group size limits the precision of pairwise comparisons involving biallelic carriers, which should therefore not be overinterpreted. Importantly, the main finding that monoallelic carriers differed biochemically from noncarriers was based on the direct comparison between these 2 groups and did not depend on the size of the biallelic group. In addition, this is a referral-based genetic testing cohort (renal and nonrenal indications), likely enriched for suspected inherited kidney disease and more extreme biochemical phenotypes; therefore, these data should not be used to infer population prevalence or absolute complication rates. The sample size is modest, reducing statistical power, particularly for subgroup analyses. Missing FEUA measurements and incomplete clinical information in some carriers may have introduced imprecision. Excluding individuals with advanced kidney disease or medications that significantly affect UA metabolism may underestimate the range of phenotypic variability. Finally, noncoding regions were not assessed, which may explain unresolved cases. Given the limited number of hard outcomes, our data support vigilance and follow-up but are not sufficient to estimate complication risk across the full spectrum of mild hypouricemia.

Despite these limitations, our findings reinforce the clinical relevance of hypouricemia, highlight overlooked carriers, and expand the allelic spectrum of renal hypouricemia. Although this cohort is from Galicia, a region with a distinctive genetic background, the observed allele-dose relationship is consistent with the conserved biology of proximal tubular urate transport. Moreover, the variants reported here show population frequencies in public resources (gnomAD; Table 2 and Supplementary Table S2), and several have been associated with serum urate levels in large-scale biobank and genome-wide association studies,^29^^,^^30^ supporting relevance beyond our region. However, variant frequencies—and the exact SUA range in which monoallelic carriers overlap noncarriers—may differ across populations, and external validation in population-based or multicenter cohorts is warranted.

Overall, renal hypouricemia should be considered a clinical spectrum in which SUA and FEUA levels reflect pathogenic allele count, and monoallelic carriers with persistent SUA between 2 and approximately 3.3 mg/dl—values frequently considered normal—may warrant FEUA assessment and longitudinal monitoring, particularly when accompanied by suggestive clinical or family history. Refining diagnostic thresholds and considering genetic testing when persistent mild hypouricemia is detected could improve renal hypouricemia diagnosis and monitoring. Recognizing gene-drug and gene-gene interactions may further refine risk stratification; future work should extend variant discovery beyond coding regions to achieve more comprehensive diagnostic yield and support personalized care.

## Disclosure

All the authors declared no competing interests.

## Supplementary Material

Supplementary File (PDF and Excel) **Supplementary Methods. Supplementary Results. Supplementary References. Figures S1.** Evolutionary conservation of amino acid residues affected by newly associated renal hypouricemia variants. **Figure S2.** Plasma and urine creatinine by pathogenic or likely pathogenic allele count. **Table S1.** Individual-level clinical, biochemical, and genetic data of study participants. **Table S2.** Variant-level evidence and ACMG/AMP classification for *SLC22A12* and *SLC2A9* variants identified in this study. STROBE Checklist.

## Footnotes

Supplementary File (PDF and Excel)

**Supplementary Methods.**

**Supplementary Results.**

**Supplementary References.**
**Figures S1.** Evolutionary conservation of amino acid residues affected by newly associated renal hypouricemia variants. **Figure S2.** Plasma and urine creatinine by pathogenic or likely pathogenic allele count. **Table S1.** Individual-level clinical, biochemical, and genetic data of study participants. **Table S2.** Variant-level evidence and ACMG/AMP classification for *SLC22A12* and *SLC2A9* variants identified in this study. STROBE Checklist.
