# Supplementary material for: Genotype-Informed Characterization of Mild Renal Hypouricemia
Source: Kidney Int Rep. 2026 Jul 22;11(10):106711. doi: 10.1016/j.ekir.2026.106711 (PMC13571878; doi:10.1016/j.ekir.2026.106711)
Supplement: Supplementary file 1 [file mmc1.pdf]

Supplementary Material:

1. **Supplementary Methods**
   - **1.1 Bioinformatics**
   - **1.2 Variant Classification According to ACMG/AMP Criteria**
   - **1.3 Impact of Pathogenic Variant Count on Uric Acid Levels: Primary and Sensitivity Analyses**
2. **Supplementary Results**
   - **2.1 Characterization of the Identified Candidate Disease-Causing Variants**
   - **2.2 Additional Statistics for Allele-Count Analyses**
   - **2.3 Sex-Stratified Analyses (Sensitivity for Consistency of the Pattern)**
   - **2.4 Age/Sex Contribution to SUA (Supporting Inclusion of Pediatric Participants)**
   - **2.5 Scatter Plot Overlay of Variant-Negative Probands**
3. **Supplementary References**
4. **Supplementary Tables**
5. **Supplementary Figures**
6. **STROBE Checklist**

1. Supplementary Methods

1.1 Bioinformatics

Descriptive statistics were computed using R version 4.4.0 and executed through RStudio version 2024.04.0 and external packages such as gt v0.11.0 (for creating descriptive tables), dplyr v1.1.4 (for data manipulation and transformation), rstatix v0.7.2 (for hypothesis testing), ggplot2 v3.5.1 (for creating graphs), and ggpubr v0.6.0 (for adding significance bars to ggplot2 charts).

Results were summarized in tables by study group. Numerical variables are reported as median (Q1, Q3) and factor variables as n/N (%).

Data preprocessing was performed using Python version 3.12.4 executed through Jupyter Notebook version 7.0.81, with the libraries NumPy v1.26.4 (for arithmetic calculations) and pandas v2.2.2 (for working with data tables). In addition to data normalization, the dimensionality of the dataset was reduced to a single clinical parameter per person to simplify the analysis, as initially two parameters per patient were available (onset and follow-up). For dimension reduction, the averages of these two values were calculated for each patient. Thus, the reported values of eGFR, SUA, FEUA, Plasma Cr, and Urine Cr in our data represent the average between the initial and follow-up measurements. If one of the values was missing, the available value was used; if both were missing, it was recorded as 'N/A' (Not Available).

1.2 Variant Classification According to ACMG/AMP Criteria

All variants were evaluated following the 2015 ACMG/AMP guidelines and subsequent ClinGen recommendations21,23, and classification was implemented using the quantitative point-based framework of Tavtigian et al.22. The following principles were applied across the variants analyzed in this study:

• **Population frequency (PM2):** Applied at moderate strength when a variant was absent or sufficiently rare in population databases for renal hypouricemia, considering disease prevalence, inheritance pattern, and available population frequencies in gnomAD v4.1.0. PM2 was not applied to p.T467M because of its elevated frequency in Roma populations, despite its established role in renal hypouricemia.

• **Recessive cases (PM3):** Applied when a variant was observed in homozygosity or in trans with another pathogenic variant in individuals with renal hypouricemia, either in our cohort or in previously published cases. The strength of PM3 was adjusted according to the number of independent observations.

• **Novel missense at same residue (PM5):** Applied at moderate strength when a different missense change affecting the same codon had previously been demonstrated as pathogenic, as in p.R171H in *SLC2A9*, given that p.R171C is a known renal hypouricemia-causing variant.

• **Segregation (PP1):** Applied when co-segregation with low SUA and/or increased FEUA was observed in our families or in previously published families. Strength was upgraded when multiple informative meioses supported segregation. For recurrent variants such as p.T467M, PP1 was applied when familial data supported co-segregation, while acknowledging incomplete penetrance or variable expressivity in heterozygous carriers.

• **Computational evidence (PP3):** Applied at supporting strength when multiple in silico predictors, including PolyPhen-2, CADD, and REVEL, together with evolutionary conservation assessed by PhyloP, supported a deleterious effect. PP3 was not applied to truncating variants, for which PVS1 was more appropriate.

• **Phenotype specificity (PP4):** Applied at supporting strength when individuals exhibited a biochemical profile highly suggestive of renal hypouricemia, particularly persistently low serum uric acid with elevated FEUA when available, and no alternative genetic or secondary cause fully explained the phenotype.

• **Functional evidence (PS3):** Applied at strong strength when functional studies directly demonstrated impaired urate transport, mislocalization of URAT1/GLUT9, or intracellular retention. To avoid redundancy, PS3 was not combined with PVS1 for truncating variants.

• **Case enrichment / population-level association evidence (PS4):** Applied cautiously, at supporting or moderate strength, when independent case enrichment in renal hypouricemia and/or strong population-level evidence from large GWAS supported an association with decreased serum urate levels. Strength was assigned according to the number of independent

cases, the robustness of the association, and whether the evidence was independent from segregation or phenotype-specific observations.

• **Predicted loss-of-function (PVS1):** Applied at very strong strength to nonsense or canonical splice-site variants in *SLC22A12* or *SLC2A9* when loss of function was considered an established disease mechanism, as illustrated by p.Y275X.

All criteria were carefully reviewed to avoid redundancy. For example, PS3 was not applied in addition to PVS1 in truncating variants, and PS4 was only used when independent case enrichment or population-level association evidence was available, not when the same information was already captured by PP1 or PP4. The final classification of each variant was determined using the point-based system proposed by Tavtigian et al.22, with Very Strong = 8 points, Strong = 4 points, Moderate = 2 points, and Supporting = 1 point; variants reaching ≥10 points were classified as pathogenic, those reaching 6–9 points as likely pathogenic, and those with insufficient evidence as variants of uncertain significance.

1.3 Impact of Pathogenic Variant Count on Uric Acid Levels: Primary and Sensitivity Analyses

For allele-count comparisons (0/1/2 pathogenic/likely pathogenic alleles), variant-negative probands were excluded and evaluated only as an exploratory overlay in the SUA–FEUA scatter plot (no genotype assignment). To minimize bias in SUA/FEUA interpretation, individuals with major urate-interpretation modifiers were excluded from allele-count comparisons (urate-modifying therapy at the time of measurement, advanced CKD affecting SUA, or coexisting genetic conditions expected to shift SUA/FEUA). Specifically, F15.S1 (thiazide therapy), F18.S1 (advanced CKD and medication; discordant SUA), F19.S1 (17q12/*HNF1B* deletion with expected opposing effect on SUA), and F24.S1 (coexisting Gitelman syndrome) were excluded from allele-count group testing (see Supplementary Table S1).

Primary group comparisons were performed in the full eligible cohort to maximize statistical power. Differences across allele-count groups were tested using Kruskal–Wallis, followed by Dunn's post hoc tests with Benjamini–Hochberg adjustment for multiple comparisons. Results were displayed using sex-stratified boxplots to assess consistency of the allele-dose pattern. Sensitivity analyses included (i) sex-stratified non-parametric testing (noting limited power in males due to small subgroup sizes), and (ii) regression-based assessment of the relative contribution of allele count, age, and sex to SUA among individuals with available age at debut.

2. Supplementary Results

2.1 Characterization of the Identified Candidate Disease-Causing Variants.

***SLC2A9* p.R171H**

The p.R171H variant in *SLC2A9* was first reported in a native Austrian family[S1], where monoallelic carriers exhibited mild hypouricemia (SUA ~2.7 mg/dL, FEUA ~14–22%), while homozygous individuals presented with severe hypouricemia (SUA 0.1 mg/dL, FEUA >150%). Another substitution affecting the same residue, p.R171C, was described in three siblings from consanguineous Israeli Arab parents, all affected by renal hypouricemia type 2 without further complications[S2]. Functional studies of that variant, p.R171C, demonstrated markedly impaired urate transport activity, reduced to ~16% of normal levels, highlighting the critical role of Arg171 in GLUT9 function[S2].

In silico classification tools initially categorized p.R171H as a variant of uncertain significance (VUS).

In our cohort, p.R171H was identified in a 12-year-old proband with suspected renal hypouricemia, presenting with serum uric acid levels below 2 mg/dL and FEUA >10% (Supplementary Table S1). Unfortunately, co-segregation analysis could not be performed.

Based on the combined evidence, including rarity in population databases, occurrence in a compatible recessive context, previous pathogenic evidence at the same residue, phenotype specificity, and prior association with renal hypouricemia, we classified SLC2A9 p.R171H as likely pathogenic according to ACMG/AMP criteria (PM2_Moderate, PM3_Supporting, PM5_Moderate, PP1_Supporting, PP4_Supporting, PS4_Supporting). Our findings add further evidence of its effect in an individual carrying a single detected *SLC2A9* variant, although additional studies are warranted to better define its monoallelic phenotypic spectrum and clinical impact.

***SLC2A9* p.T125M**

The p.T125M variant in *SLC2A9* was first described in homozygosity in a Sephardic Jewish patient with renal hypouricemia and was later identified in multiple Spanish families, where haplotype analysis demonstrated a founder effect[S2,S3]. This variant represents the most frequent cause of renal hypouricemia type 2 in Spain, accounting for over 90% of reported cases, with homozygous carriers showing severe hypouricemia and monoallelic carriers typically displaying milder reductions in serum uric acid (SUA).

Functional studies in *Xenopus* oocytes demonstrated that p.T125M markedly reduces GLUT9 urate transport capacity due to decreased expression of the mutant protein[S2]. Comparative data

indicate that homozygous carriers of p.T125M present with more profound hypouricemia than those with the common *SLC22A12* p.T467M variant, reinforcing the critical role of GLUT9 in urate reabsorption.

In silico predictions suggest a potential deleterious effect: PolyPhen-2 (0.932) and CADD (28.2) predict a damaging impact, and PhyloP (6.81) indicates strong evolutionary conservation of the affected residue. REVEL provides an uncertain/intermediate score (0.519), which does not clearly support a damaging effect. Pathogenicity is further supported by the low population frequency of the variant (PopMax in GnomAD v4.1.0: MAF ~7.75×10^{-4}).

In our cohort, p.T125M was identified in three monoallelic probands (F15.S1, F24.S1, and F26.S1; Supplementary Table S1). Proband F26.S1 presented with markedly reduced SUA levels (1.7–1.9 mg/dL) and elevated FEUA (22.9%). In proband F15.S1, hypouricemia was milder: her earliest available records, obtained at age 52 while she was already receiving thiazide therapy for hypercalciuria, showed SUA 2.8 mg/dL, uricosuria 39.6 mg/dL, and FEUA 12%. Later measurements at ages 54 and 57 showed slightly higher SUA values (3.7 and 3.6 mg/dL) and lower FEUA (9.3% and 6.9%). Because thiazides can increase SUA by reducing urate excretion and promoting proximal tubular urate reabsorption, these measurements may not reflect baseline urate handling and could partially mask the urate-lowering effect associated with the variant. Proband F24.S1 (SUA 2.7–3.0 mg/dL) also carried two pathogenic variants in *SLC12A3* causing Gitelman syndrome. Given the coexisting Gitelman syndrome, her uric acid levels may reflect the combined effects of both genetic conditions and should therefore be interpreted with caution.

Based on the combined functional, clinical, genetic, and population-level evidence, including rarity, supportive computational predictions, phenotype specificity, published functional studies, segregation/clinical evidence, and previous enrichment in affected individuals, we classified *SLC2A9* p.T125M as pathogenic according to ACMG/AMP criteria (PM2_Moderate, PP1_Supporting, PP3_Supporting, PP4_Supporting, PS3_Strong, PS4_Moderate). This variant is a well-established cause of renal hypouricemia type 2, with homozygous carriers developing severe hypouricemia and monoallelic carriers showing variable, generally milder phenotypes that may be influenced by additional genetic or treatment-related factors.

SLC22A12 p.V138L

The p.V138L variant (c.412G>C, rs149722479) in *SLC22A12* was first reported by Claverie-Martin Laboratory in a patient with hypouricemia, where it was found in trans with another pathogenic variant, and was subsequently submitted to ClinVar (SCV001745893.1). Population-level evidence further supports its effect on urate handling, as p.V138L was

significantly associated with decreased serum urate levels in a large GWAS meta-analysis[29] ($\beta = -1.69$, $p = 8.2 \times 10^{-65}$). In addition, codon 138 appears to be functionally relevant for URAT1 activity, as a different substitution affecting the same residue, p.V138M, was previously identified in patients with renal hypouricemia and functionally characterized as impairing URAT1-mediated urate transport[24].

In silico predictions also suggest a potential deleterious effect: PolyPhen-2 (0.999) and CADD (24.7) strongly predict a damaging impact, while REVEL provides moderate support (0.567). PhyloP indicates strong evolutionary conservation of this residue (4.62), reinforcing its potential functional relevance.

In our cohort, p.V138L was identified in two probands. The first was a homozygous child (F17.S1), diagnosed with renal hypouricemia during neonatal follow-up for prematurity and with a family history of kidney stones. The second was a monoallelic 76-year-old woman (F18.S1) with a long-standing history of kidney stones since early adulthood and renal cysts detected by ultrasound, who has been under nephrology follow-up for hypouricemia during the past five years. The earliest electronic laboratory record available to us, obtained when she was 59 years old, showed no hypouricemia (SUA 4.3 mg/dL). Hypouricemia was first documented in a later test, when she was 71 years old. Because we did not have access to her complete historical laboratory records, we could not determine the temporal pattern of her uric acid levels. Therefore, it remains unclear whether the SUA value of 4.3 mg/dL represented an isolated finding, whether hypouricemia had been present intermittently or earlier in life, or whether it was triggered later by external factors.

Based on the combined evidence, including rarity, occurrence in a compatible recessive context, supportive computational predictions, phenotype specificity, functional relevance of the affected residue, and previous association/enrichment evidence, we classified *SLC22A12* p.V138L as likely pathogenic according to ACMG/AMP criteria (PM2_Moderate, PM3_Supporting, PP3_Supporting, PP4_Supporting, PS4_Moderate). Our findings support a pathogenic effect in the homozygous state, while the clinical impact of monoallelic p.V138L remains inconclusive.

***SLC22A12* p.L144P**

The p.L144P (rs148915713) variant in *SLC22A12* was first reported in 2015 in an international cohort of 272 typical kidney stone formers screened for variants in 30 genes implicated in nephrolithiasis and nephrocalcinosis[S4]. In that study, it was identified in monoallelic state in a single patient presenting with nephrolithiasis and hypouricemia, with no additional causative variants detected. In addition, a large genome-wide association study reported a highly

significant association between p.L144P and decreased serum urate levels[29] ($\beta = -1.66$, SE = 0.06, $p = 8.8 \times 10^{-165}$), supporting an effect of this variant on urate metabolism.

In silico predictions consistently support a deleterious effect: REVEL = 0.869, PolyPhen-2 = 0.999, and CADD = 24.7. Conservation data indicate moderate evolutionary conservation of the affected residue (PhyloP = 2.76).

In our renal hypouricemia probands group, the variant was detected in heterozygous/monoallelic state in a 3-year-old proband (F06.S1) and his twin sister (F06.S3), both showing persistent mild hypouricemia (SUA ~2 mg/dL) with elevated FEUA. Their mother (F06.S2), who did not carry the variant, had higher SUA levels (Figure 2, Supplementary Table S1). Screening of the remaining cohort identified eight additional carriers. Clinical and biochemical data were available for one of them, F31.S1 from the RENAL group, a 69-year-old woman recently referred for suspected glomerulopathy. Medical records dating back to age 53 showed creatinine 0.8 mg/dL and sUA 2.7 mg/dL, compatible with mild hypouricemia, although FEUA was not documented. Over time, renal function declined substantially, accompanied by rising uric acid levels in parallel with the decrease in glomerular filtration. Genetic analysis using a 400-gene renal panel identified p.L144P in *SLC22A12* and a heterozygous pathogenic variant in *LMNA*, with no other causative variants detected.

Based on the combined evidence, including rarity, segregation/clinical evidence, supportive computational predictions, phenotype specificity, previous report in a patient with nephrolithiasis and hypouricemia, and strong population-level association with decreased serum urate, we classified *SLC22A12* p.L144P as likely pathogenic according to ACMG/AMP criteria (PM2_Moderate, PP1_Supporting, PP3_Supporting, PP4_Supporting, PS4_Moderate). The compatible biochemical findings in our monoallelic carriers support an effect of this variant on urate handling, although its direct contribution to renal function decline in F31.S1 cannot be determined from the available data.

SLC22A12 p.P268S

The p.P268S variant in *SLC22A12* is extremely rare, reported in only two individuals among 804,985 in gnomAD v4.1.0, including one individual from the non-Finnish European population. To our knowledge, it has not been previously associated with renal hypouricemia. The affected residue is highly conserved, and predictive tools support a deleterious effect: PolyPhen-2 (0.965), CADD (22.9) and REVEL (0.664) indicate a damaging or potentially damaging impact, while PhyloP (6.67) supports strong evolutionary conservation. Automated classifiers such as VarSome initially categorized it as a variant of uncertain significance (VUS).

In our cohort, p.P268S was identified in proband F11.S1, a 3-year-old female with microscopic hematuria and occasional voiding symptoms. Laboratory studies revealed mild hypouricemia (sUA 2.3–2.9 mg/dL) with elevated FEUA (13.3%). Other laboratory results were largely normal, except for intermittently high-normal calciuria. Genetic testing, using a panel of 400 genes related to the most prevalent hereditary kidney diseases, detected only the p.P268S variant in the *SLC22A12*. Co-segregation analysis was performed in her sister (F11.S4), parents (F11.S2, F11.S3), maternal aunt (F11.S6), and maternal grandmother (F11.S5). The genetic variant co-segregated with urate abnormalities within the family: all carriers (F11.S2 and F11.S4) showed significantly lower SUA and increased FEUA compared with non-carriers (F11.S3, F11.S5, and F11.S6) (Figure 3, Supplementary Table S1).

Screening the remaining cohort identified six additional carriers of this variant, including two individuals from our RENAL group: F27.S1 and her father, both affected by *COL4A4*-associated disease. Clinical data showed that F27.S1, a 37-year-old woman, consistently had SUA values < 3 mg/dL, compatible with mild renal hypouricemia, although FEUA data were not available. Her father could not be assessed because of current medication and lack of previous biochemical data. No clinical information was available for the remaining carriers.

Based on the combined evidence, including extreme rarity, conservation of the affected residue, concordant *in silico* predictions, co-segregation with altered urate handling in family F11, phenotype specificity, and observation of an additional carrier with persistent low SUA, we classified *SLC22A12* p.P268S as likely pathogenic according to ACMG/AMP criteria (PM2_Moderate, PP1_Supporting, PP3_Supporting, PP4_Supporting, PS4_Supporting). We therefore propose p.P268S as a novel *SLC22A12* variant associated with renal hypouricemia.

***SLC22A12* p.Y275X**

The p.Y275X variant is a truncating variant that, to our knowledge, has not been previously observed in population databases including gnomAD (v4.1.0) or in our Galician cohort, suggesting that it is extremely rare or possibly *novel*. Although no functional study has been conducted for this specific variant, other truncating variants in *SLC22A12* have been established as pathogenic and associated with renal hypouricemia, supporting loss of function as a recognized disease mechanism for this condition[7,24, 25].

In our cohort, p.Y275X was identified in a female patient (F19.S1) with CAKUT and hypomagnesemia associated with a known and recurrent deletion involving *HNF1B*. Biochemical data showed serum uric acid levels at the lower limit of the normal range (3.7 and 3.9 mg/dL), and no FEUA records were available. These findings do not allow a definitive assessment of the biochemical effect of p.Y275X in this patient. However, because *HNF1B*-related disease is typically associated with increased uric acid levels[26], one possible

interpretation is that the patient's low-normal SUA values may reflect opposing effects of the *HNF1B* deletion and the truncating *SLC22A12* variant. Under this hypothesis, the urate-lowering effect expected from p.Y275X could be partially counterbalanced by the hyperuricemic tendency associated with *HNF1B* disease. Further functional and clinical evidence will be required to confirm the effect of p.Y275X on URAT1 function and its contribution to renal hypouricemia.

Based on its absence or extreme rarity in population databases and its predicted loss-of-function effect in *SLC22A12*, a gene in which loss of function is an established mechanism of renal hypouricemia, we classified *SLC22A12* p.Y275X as pathogenic according to ACMG/AMP criteria (PVS1_VeryStrong, PM2_Moderate). Although the biochemical effect of this specific variant could not be definitively assessed in our patient because of the coexisting *HNF1B*/17q12 deletion, the genetic evidence strongly supports pathogenicity.

***SLC22A12* p.S282L**

The p.S282L variant in *SLC22A12* has not been previously associated with renal hypouricemia and is currently listed in ClinVar as a VUS (one-star review status). It is extremely rare in population databases (gnomAD v4.1.0: MAF = 1.6×10^{-5} in non-Finnish Europeans; PopMax = 9.0×10^{-5}).

In silico predictors consistently support a deleterious effect: PolyPhen-2 (0.977, "probably damaging"), CADD (23.8, above the pathogenicity threshold), and REVEL (0.640, moderate-to-high impact). Conservation analysis (PhyloP = 4.85) also indicates that the affected residue is highly conserved (PhyloP = 4.85).

In our cohort, the variant was identified in proband F08.S1, a 4-year-old female presenting with pseudohypoaldosteronism, hypouricemia, and hyperuricosuria. Genetic testing revealed a pathogenic *NR3C2* variant explaining pseudohypoaldosteronism, together with the *SLC22A12* p.S282L variant, which was initially classified as a VUS by automated classification platforms such as VarSome or Franklin. Her mother (F08.S2), also carrying both variants, exhibited persistent hypouricemia, supporting co-segregation of p.S282L with urate abnormalities within this family. An additional carrier from the RENAL group (F28.S1), a 50-year-old male, showed consistent hypouricemia (SUA <2 mg/dL) and elevated FEUA (17%), compatible with previously unrecognized renal hypouricemia. Screening of broader cohorts revealed five more carriers from the RELATIVES, RENAL, and NON-RENAL groups, although no clinical data were available for these individuals.

Based on the combined evidence, including rarity, supportive computational predictions, high evolutionary conservation, co-segregation with urate abnormalities in family F08, phenotype

specificity, and independent observation of a compatible biochemical phenotype in F28.S1, we classified *SLC22A12* p.S282L as likely pathogenic according to ACMG/AMP criteria (PM2_Moderate, PP1_Supporting, PP3_Supporting, PP4_Supporting, PS4_Supporting). We therefore propose p.S282L as a novel *SLC22A12* variant associated with renal hypouricemia.

***SLC22A12* p.R434C**

The p.R434C variant in *SLC22A12* was reported by Tasic et al.[S5] in two unrelated Macedonian families. The two probands, both monoallelic for the variant, were a 7-year-old with nephrolithiasis, hypouricemia (SUA 0.72–1.18 mg/dL), and elevated FEUA (20.8%), and a 5-year-old with hypouricemia (SUA 1.24 mg/dL), severe dehydration, and nephrocalcinosis. In the first family, the father and brother were also heterozygous: the father had normal urate levels (although no exact reference value for normal urate was given), whereas the brother showed reduced serum urate (1.56 mg/dL). In the second family, the mother was heterozygous, with SUA 2.3 mg/dL, elevated FEUA (19.3%), and a history of renal colic with passage of a calculus, consistent with a mild renal hypouricemia phenotype. Functional studies in HEK293 cells confirmed reduced membrane expression and markedly impaired urate transport[5].

More recently, large-scale genome-wide association studies have identified this variant as significantly associated with decreased serum urate levels[29] ($\beta = -1.41$, $p = 2.7 \times 10^{-19}$; $\beta = -0.69$, $p = 2.0 \times 10^{-14}$), further supporting its impact on urate metabolism.

In silico prediction algorithms provide mixed evidence: while PolyPhen-2 and REVEL classify the variant as probably benign or of uncertain significance, CADD (20.8) indicates possible deleteriousness. PhyloP highlights high conservation of the affected residue, suggesting functional importance despite discordant computational predictions.

In our cohort, p.R434C was detected in proband F14.S1, who was monoallelic for the variant and presented with SUA levels of 1.7–2.1 mg/dL and FEUA of 21.9%, consistent with values previously reported for some monoallelic carriers. We also identified 11 additional carriers, including two individuals from the RENAL group with available biochemical data. Proband F29.S1, a 38-year-old female, showed persistent SUA levels between 2.5 and 3 mg/dL since age 22, consistent with mild hypouricemia, although FEUA data were not available. Similarly, F30.S1, a 42-year-old woman, had an initial SUA value of 3 mg/dL at age 22, with subsequent values remaining stable (2.9 mg/dL at age 41), also compatible with mild hypouricemia. The absence of FEUA data in these two cases prevents definitive biochemical characterization.

Based on the combined genetic, functional, population-level, and clinical evidence, including rarity, segregation/clinical evidence, phenotype specificity, published functional studies showing impaired URAT1-mediated urate transport, and previous association/enrichment evidence, we classified *SLC22A12* p.R434C as pathogenic according to ACMG/AMP criteria

(PM2_Moderate, PP1_Supporting, PP4_Supporting, PS3_Strong, PS4_Moderate). The published cases and our observations indicate that monoallelic p.R434C can be associated with biochemical profiles ranging from classical to mild renal hypouricemia, although additional studies are required to define penetrance and complication risk in heterozygous carriers.

SLC22A12 p.T467M

The p.T467M variant in *SLC22A12* has been consistently associated with hypouricemia across numerous studies. It is one of the two major variants causing renal hypouricemia type 1 in Roma populations from the Czech Republic, Slovakia, and Spain, where it reaches relatively high frequencies of 1.9%–5.6%[S6–S8]. This variant has been linked to severe manifestations in homozygous or compound heterozygous carriers[S6–S8] and to milder phenotypes in monoallelic individuals[8].

Moreover, large-scale genome-wide association studies have reported a significant association between p.T467M and decreased serum urate levels[29] ($\beta = -1.23$; $p = 8.6 \times 10^{-25}$), further supporting its contribution to altered urate handling.

In silico predictions provided mixed evidence: PolyPhen-2 and CADD suggest a damaging impact, and the strong evolutionary conservation indicated by the PhyloP score supports the functional relevance of this residue; however, the REVEL score (0.367) is below commonly used deleteriousness thresholds and does not support a damaging effect. Accordingly, computational evidence for p.T467M was considered supportive only in the context of the broader *in silico* profile and was not interpreted as uniformly concordant. Experimental data corroborates its pathogenicity, demonstrating impaired urate uptake, mislocalization of the URAT1 protein, and retention within the endoplasmic reticulum[7]. Despite this evidence, automated classification tools such as VarSome and Franklin categorize the variant as benign, largely because of its relatively high frequency in specific populations.

In our cohort, p.T467M was detected in three renal hypouricemia probands (F01.S1, F05.S1, and F16.S1) with variable clinical presentations. The homozygous carriers (F01.S1 and F05.S1) showed severe hypouricemia (SUA < 2 mg/dL), consistent with previous observations in biallelic *SLC22A12* carriers. In family F05, segregation data were available for four relatives: three heterozygous carriers (F05.S2, F05.S3, and F05.S4) and one non-carrier (F05.S5). Unexpectedly, the monoallelic carriers did not show reduced or mildly reduced SUA levels, in contrast to other monoallelic carriers in our cohort, while the non-carrier had normal SUA levels. By contrast, proband F16.S1, who was also heterozygous for p.T467M, showed moderate hypouricemia (SUA ~3 mg/dL), consistent with previous reports of mild phenotypes in monoallelic carriers but differing from the heterozygous relatives in family F05.

Based on the combined evidence, including occurrence in affected individuals in a compatible recessive context, segregation/clinical evidence, supportive computational predictions, phenotype specificity, published functional studies showing impaired urate uptake and URAT1 mislocalization, and previous association/enrichment evidence, we classified *SLC22A12* p.T467M as pathogenic according to ACMG/AMP criteria (PM3_Supporting, PP1_Supporting, PP3_Supporting, PP4_Supporting, PS3_Strong, PS4_Moderate). Our findings support its pathogenicity, particularly in homozygous carriers, while also highlighting incomplete penetrance and variable expressivity in heterozygous individuals.

***SLC22A12* p.A476D**

The p.A476D variant in *SLC22A12* has previously been reported in a Spanish renal hypouricemia cohort[S9], where it was identified in two affected members of the same family as monoallelic carriers. Functional analysis performed in that study using *Xenopus* showed that the c.1427C>A substitution reduces urate transport without altering URAT1 localization at the plasma membrane[S9].

In silico predictions provide additional support: PolyPhen-2 and CADD classify the variant as potentially damaging, while REVEL introduces uncertainty by suggesting it is less likely to be deleterious. PhyloP indicates moderate conservation, supporting the possible functional importance of this residue.

In our renal hypouricemia probands group, p.A476D was detected in the monoallelic state in a male patient (F09.S1) referred at age 13 for episodes of tenesmus, increased urinary frequency, and nocturia, during which hypercalciuria was diagnosed. Biochemical evaluation revealed mild hypouricemia (SUA 2.2–2.4 mg/dL) with inappropriately elevated FEUA (12.3% and 11.3%), while the rest of the clinical study was unremarkable. Family history was notable for nephrolithiasis in the maternal grandparents. No additional pathogenic or likely pathogenic variants were detected in the 400 genes analyzed by the genetic panel, and no other carriers of this variant were identified in the RENAL or NON-RENAL groups.

Based on the combined evidence, including rarity, phenotype specificity, and published functional evidence showing reduced URAT1-mediated urate transport, we classified *SLC22A12* p.A476D as likely pathogenic according to ACMG/AMP criteria (PM2_Moderate, PP4_Supporting, PS3_Strong). The available evidence supports an effect on urate handling, including in the monoallelic state, although further studies are needed to better define its penetrance, phenotypic spectrum, and clinical relevance.

2.2 Additional Statistics for Allele-Count Analyses.

Across the full eligible cohort, SUA differed significantly by pathogenic allele count (Kruskal–Wallis $H = 13.83$, $p = 9.96\times10^{-4}$; SUA available for 0/1/2 alleles: n = 5/18/3). Dunn's post hoc tests (Benjamini–Hochberg adjusted) showed lower SUA in monoallelic carriers versus non-carriers (p_adj = 0.0197) and lower SUA in biallelic carriers versus monoallelic carriers (p_adj = 0.0220); the 0 vs 2 comparison was also significant (p_adj = 7.22×10^{-4}). Median SUA values were 4.50 (0 alleles), 2.55 (1 allele), and 0.80 mg/dL (2 alleles), supporting an allele-dose effect.

FEUA also differed by allele count (Kruskal–Wallis $H = 10.23$, $p = 6.02\times10^{-3}$; FEUA available for 0/1/2 alleles: n = 3/11/3). Dunn's post hoc tests (Benjamini–Hochberg adjusted) supported higher FEUA in biallelic carriers versus non-carriers (p_adj = 0.00416). Differences between adjacent groups reached borderline significance in this dataset (0 vs 1: p_adj = 0.0494; 1 vs 2: p_adj = 0.0494), but interpretation is limited by incomplete FEUA availability.

2.3 Sex-Stratified Analyses (Sensitivity for Consistency of the Pattern)

In females, the allele-dose association remained significant for SUA (Kruskal–Wallis $H = 11.00$, $p = 4.09\times10^{-3}$; n = 4/14/2 for 0/1/2 alleles) with post hoc differences in the expected direction (Dunn BH: 0 vs 1 p_adj = 0.0428; 0 vs 2 p_adj = 0.0164). FEUA in females also differed by allele count ($H = 7.73$, $p = 0.0210$; n = 2/8/2), driven primarily by the contrast between 0 and 2 alleles (Dunn BH: p_adj = 0.0164).

In males, the same monotonic pattern was observed (median SUA: 5.10 → 2.30 → 0.80 mg/dL for 0/1/2 alleles; median FEUA: 11.8 → 15.45 → 41.55%), but the analysis was considered non-informative because subgroup sizes were very small, particularly for non-carriers and biallelic carriers (SUA n = 1/3/1; FEUA n = 1/3/1).

2.4 Age/Sex Contribution to SUA (Supporting Inclusion of Pediatric Participants)

Among individuals with SUA and debut age available (n = 21), SUA was most strongly associated with pathogenic allele count. In an adjusted linear model (SUA ~ allele count + sex + debut age), allele count remained significant ($\beta = -1.51$ mg/dL per additional pathogenic allele, $p = 6.20\times10^{-4}$), whereas debut age ($p = 0.72$) and sex ($p = 0.92$) were not significant predictors. Allele count increased model fit substantially beyond age and sex ($\Delta R^2 \approx 0.39$). In adults-only model (debut age ≥18 years; n = 9 with SUA), allele count remained associated with SUA ($\beta = -1.46$ mg/dL per allele, $p = 0.041$), although interpretation is limited by the small number of adult biallelic carriers.

2.5 Scatter Plot Overlay of Variant-Negative Probands

For the SUA–FEUA scatter plot, paired SUA–FEUA data were available for 22 individuals, including non-carriers (n = 3), monoallelic carriers (n = 11), biallelic carriers (n = 3), and

variant-negative probands (n = 5). Variant-negative probands were overlaid as an independent exploratory group and were not used for allele-count statistical testing.

3. Supplementary References

S1. Windpessl M, Ritelli M, Wallner M, Colombi M. A novel homozygous SLC2A9 mutation associated with renal-induced hypouricemia. *Am J Nephrol*. 2016;43(4). doi:10.1159/000445845

S2. Dinour D, Gray NK, Ganon L, et al. Two novel homozygous SLC2A9 mutations cause renal hypouricemia type 2. *Nephrology Dialysis Transplantation*. 2012;27(3). doi:10.1093/ndt/gfr419

S3. Perdomo-Ramirez A, Cordoba-Lanus E, Trujillo-Frias CJ, et al. Pathogenic Variants of SLC22A12 (URAT1) and SLC2A9 (GLUT9) in Spanish Patients with Renal Hypouricemia: Founder Effect of SLC2A9 Variant c.374C>T; p.(T125M). *Int J Mol Sci*. 2023;24(9). doi:10.3390/ijms24098455

S4. Halbritter J, Baum M, Hynes AM, et al. Fourteen monogenic genes account for 15% of nephrolithiasis/nephrocalcinosis. *Journal of the American Society of Nephrology*. 2015;26(3). doi:10.1681/ASN.2014040388

S5. Tasic V, Hynes AM, Kitamura K, et al. Clinical and functional characterization of URAT1 variants. *PLoS One*. 2011;6(12). doi:10.1371/journal.pone.0028641

S6. Stiburkova B, Gabrikova D, Čepek P, et al. Prevalence of URAT1 allelic variants in the Roma population. *Nucleosides Nucleotides Nucleic Acids*. 2016;35(10-12). doi:10.1080/15257770.2016.1168839

S7. Stiburkova B, Sebesta I, Ichida K, et al. Novel allelic variants and evidence for a prevalent mutation in URAT1 causing renal hypouricemia: Biochemical, genetics and functional analysis. *European Journal of Human Genetics*. 2013;21(10). doi:10.1038/ejhg.2013.3

S8. Gabrikova D, Bernasovska J, Sokolova J, Stiburkova B. High frequency of SLC22A12 variants causing renal hypouricemia 1 in the Czech and Slovak Roma population; simple and rapid detection method by allele-specific polymerase chain reaction. *Urolithiasis*. 2015;43(5). doi:10.1007/s00240-015-0790-4

S9. Claverie-Martin F, Trujillo-Suarez J, Gonzalez-Acosta H, et al. URAT1 and GLUT9 mutations in Spanish patients with renal hypouricemia. *Clinica Chimica Acta*. 2018;481. doi:10.1016/j.cca.2018.02.030

4. Supplementary Tables

Supplementary Table S1. Individual-Level Clinical, Biochemical, and Genetic Data of Study Participants. Group: Study subgroup: renal hypouricemia probands, relatives, renal group, or non-renal group. **Subject ID**: Unique identifier of each participant. **Relationship**: Relationship to the proband within each family, when applicable. **Sex**: Biological sex (M/F). **Debut age (years)**: Age at the first documented low serum uric acid measurement or first evaluation in which hypouricemia was identified, or to the first available biochemical evaluation for relatives or non-carriers. Hypouricemia may have been present before first detection. **Follow-up duration (years)**: Time between debut and the most recent available evaluation. **FH:** Family history of relevant findings, including hypouricemia, hyperuricosuria, nephrolithiasis, voiding symptoms, or hematuria, as recorded in clinical files (Y = yes; blank/NA = not available). **Genotypic status (*SLC22A12*/*SLC2A9*)**: Classification based only on *SLC22A12* and *SLC2A9*: biallelic, monoallelic, variant-negative, or non-carrier. Variant-negative indicates no pathogenic/likely pathogenic variant identified in these genes. **Gene**: Gene carrying the pathogenic/likely pathogenic variant. **Variant (protein)**: Protein-level variant identified in *SLC22A12* or *SLC2A9*. **Zygosity**: Zygosity of the pathogenic/likely pathogenic variant. Blank/NA indicates that the field was not applicable. **Presentation/referral**: Main reason for referral or clinical context. **Urate interpretation modifiers/exclusions**: Key factors potentially modifying SUA/FEUA interpretation or leading to exclusion from quantitative allele-count analyses, such as advanced CKD, discordant SUA, urate-modifying medication, or additional genetic findings including *HNF1B*/17q12 deletion or Gitelman syndrome. **Urate-modifying medication**: Medications with recognized effects on serum uric acid, such as thiazides, which increase SUA, or losartan, which decreases SUA; NA if none were recorded. **SUA_Onset / SUA_Follow-up (mg/dL)**: Serum uric acid at debut and at the most recent available evaluation. **FEUA_Onset / FEUA_Follow-up (%)**: Fractional excretion of uric acid at debut and follow-up. **PlasmaCr_Onset / PlasmaCr_Follow-up (mg/dL)**: Plasma creatinine at debut and follow-up. **UrineCr_Onset / UrineCr_Follow-up (mg/dL)**: Urine creatinine at debut and follow-up. Missing values indicate that measurements were not available in the clinical record. **Renal outcome**: Renal events recorded during follow-up, including AKI, nephrolithiasis, CKD, or other renal complications. “None reported” indicates no renal complications documented in the available data. **Assay (gene count; platform; year)**: Genetic test used, including panel, WES, or targeted Sanger testing; number of genes assessed; sequencing platform; and year performed. All assays included *SLC22A12* and *SLC2A9*. **Sequenced urate-handling genes beyond *SLC22A12*/*SLC2A9***: Additional urate-handling genes covered by each assay; “genome-wide” for WES; NA for targeted Sanger testing.

Supplementary Table S2. Variant-Level Evidence and ACMG/AMP Classification for *SLC22A12* and *SLC2A9* Variants Identified in This Study.

Gene: Gene in which the variant was identified. **Transcript:** RefSeq transcript used for HGVS nomenclature. **Variant (GRCh38):** Chromosome:position in GRCh38/hg38, followed by the reference and alternative allele. **Exon:** Exon number according to the indicated transcript. **Variant type:** Variant consequence, such as missense or null variant. **HGVS cDNA:** HGVS cDNA nomenclature. **HGVS protein:** HGVS protein nomenclature. **dbSNP:** Reference SNP identifier, when available. **gnomAD NFE MAF (v4.1.0):** Minor allele frequency in the non-Finnish European population in gnomAD v4.1.0. **gnomAD PopMax (v4.1.0):** Highest minor allele frequency observed in any gnomAD population in v4.1.0. **Probands (n) / Relatives (n) / Renal group (n) / Non-renal group (n):** Number of carriers identified in each study subgroup. Values in parentheses indicate the number of carriers with available biochemical data, when shown. The renal group includes individuals tested for kidney diseases other than suspected renal hypouricemia; the non-renal group includes individuals referred for non-renal genetic conditions whose sequencing included *SLC22A12* and *SLC2A9*. **Other affected carriers:** Whether additional carriers outside probands had clinical or biochemical evidence consistent with renal hypouricemia (Y = yes; N = no; N/A = not evaluable). **Segregation:** Segregation consistent with altered urate handling observed in our families and/or in previously published families (Y = yes; N = no; N/A = not evaluable). For variants showing variable expressivity in heterozygotes, such as *SLC22A12* p.T467M, segregation evidence may coexist with incomplete penetrance of the monoallelic biochemical phenotype. **Previously linked to renal hypouricemia:** Whether the variant had previously been associated with renal hypouricemia in the literature (Y = yes; N = no). **Key reference(s) (PMID):** PubMed identifier(s) supporting prior association and/or key evidence. GWAS PMIDs, when shown, refer to population-level associations with serum uric acid levels. **PolyPhen-2 (max) / CADD / REVEL / PhyloP:** *In silico* predictors provided as supportive computational evidence. For variants with mixed computational evidence, including *SLC22A12* p.T467M and *SLC2A9* p.T125M, PP3 was applied only at supporting strength after considering the overall *in silico* profile; REVEL scores below or within the uncertain range were explicitly considered and were not interpreted as independently supporting a deleterious effect. **VarSome / Franklin:** Default automated classification returned by VarSome and Franklin, respectively. **ClinVar (stars):** ClinVar classification and review status, when available. **Authors' classification (ACMG/AMP):** Final variant classification assigned by the authors according to ACMG/AMP guidelines after reviewing all available evidence; the applied ACMG/AMP criteria are listed in parentheses. **ACMG/AMP criteria abbreviations used in the table:** PVS1, null variant in a gene where loss of function is a known mechanism of disease; PS3, functional studies; PS4, increased prevalence in affected individuals; PM2, rarity in population databases; PM3, allelic data in recessive disorders; PM5, novel amino acid change at a residue with a different

established pathogenic change; PP1, segregation; PP3, computational evidence; PP4, phenotype specificity; PP5, reputable source.

5. Supplementary Figures

Supplementary Figure S1. Evolutionary Conservation of Amino Acid Residues Affected by Newly Associated Renal Hypouricemia Variants. Sequence alignment of the URAT1, encoded by *SLC22A12*, is shown for human and selected orthologous vertebrate species. Residues affected by the three *SLC22A12* variants newly associated with renal hypouricemia in this study (p.S282L, p.P268S, and p.Y275X) are highlighted in yellow. All three residues show strong evolutionary conservation across species.

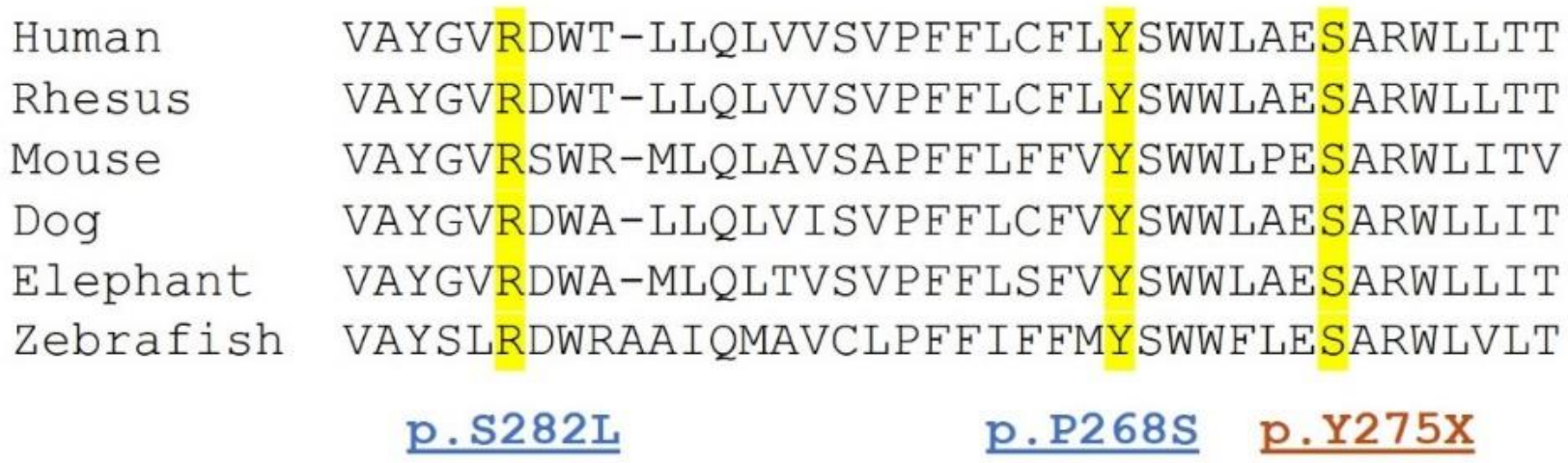

Supplementary Figure S2. Plasma and Urine Creatinine by Pathogenic/Likely Pathogenic Allele Count. Box plots comparing plasma creatinine (a) and urine creatinine (b) (mg/dL) across individuals with 0, 1, or 2 pathogenic/likely pathogenic alleles in *SLC22A12* or *SLC2A9*. Variant-negative probands were excluded from allele-count comparisons. Individuals with predefined clinical or biochemical modifiers affecting urate interpretation were excluded from allele-count comparisons, as described in the Supplementary Methods and Supplementary Table S1. Data are shown stratified by sex. Group differences were assessed using Kruskal–Wallis tests followed by Dunn's post hoc comparisons, with multiple-testing correction as specified in the Supplementary Methods. No statistically significant differences were observed between allele-count groups.

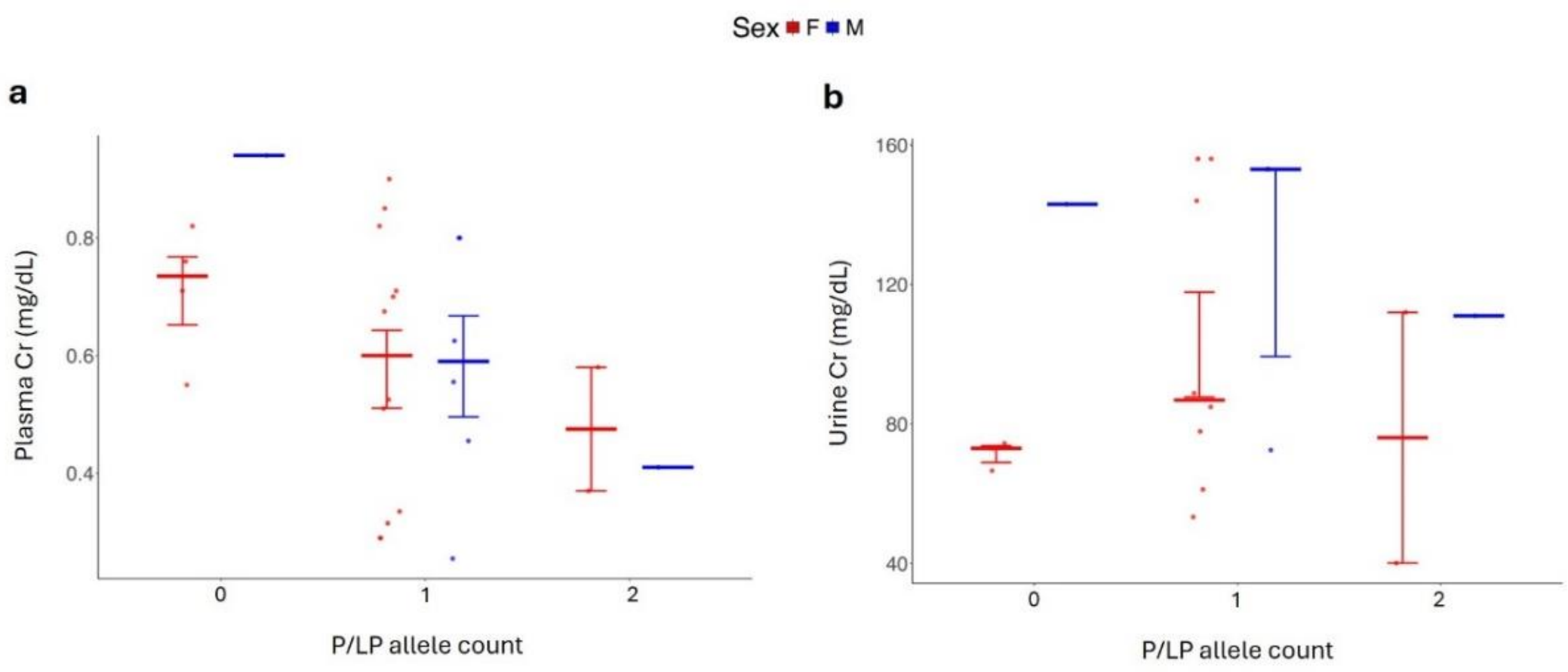

6. STROBE Checklist

STROBE Statement—checklist of items that should be included in reports of observational studies

	Item No.	Recommendation	Page No.	Relevant text from manuscript
**Title and abstract**	1	(*a*) Indicate the study's design with a commonly used term in the title or the abstract	3	"This study evaluated genotype–phenotype correlations across individuals with 0, 1, or 2 pathogenic alleles in a referral-based genetic testing cohort from Galicia (Western Europe)." (Abstract – Introduction)
		(*b*) Provide in the abstract an informative and balanced summary of what was done and what was found	3	The Abstract includes structured Introduction, Methods, Results, and Conclusions, summarizing the rationale, study groups, genotype–phenotype analyses, diagnostic yield, allele-dose pattern, and clinical implications.
**Introduction**				
Background/rationale	2	Explain the scientific background and rationale for the investigation being reported	3-5	"Renal hypouricemia is caused by pathogenic variants in SLC22A12 and SLC2A9." "Current diagnostic thresholds based on serum uric acid (SUA <2 mg/dL) and fractional excretion of uric acid (FEUA >10%) may overlook individuals with monoallelic variants and mild hypouricemia." (Abstract and Introduction)
Objectives	3	State specific objectives, including any prespecified hypotheses	3, 5	"This study evaluated genotype–phenotype correlations across individuals with 0, 1, or 2 pathogenic alleles…" (Abstract). "Here, we investigated the genetic and clinical features of renal hypouricemia in a referral-based cohort from Galicia… focused on individuals with mild hypouricemia, explored overlooked cases… and evaluated genotype–phenotype correlations across pathogenic allele counts." (Introduction)
**Methods**				

Study design	4	Present key elements of study design early in the paper	3, 5,6	"We analyzed probands referred for suspected renal hypouricemia, their relatives, and additional carriers not previously suspected of the condition. Genetic, biochemical, and clinical data were integrated to characterize SUA and FEUA distributions by genotype and to identify overlooked cases." (Abstract – Methods; Methods – Study Participants and Recruitment)
Setting	5	Describe the setting, locations, and relevant dates, including periods of recruitment, exposure, follow-up, and data collection	5-7	"This study used the NephroGalGen cohort, a referral-based genetic testing cohort comprising approximately 5,400 individuals referred between 2013 and 2025 to either NefroCHUS or the Fundación Pública Galega de Medicina Xenómica (FPGMX) for renal and non-renal indications." "Both laboratories receive samples from the 12 public hospitals across Galicia…" (Methods – Study Participants and Recruitment). Biochemical values were recorded at Onset and Follow-up when available (Methods – Clinical Characterization).
Participants	6	(*a*) *Cohort study*—Give the eligibility criteria, and the sources and methods of selection of participants. Describe methods of follow-up *Case-control study*—Give the eligibility criteria, and the sources and methods of case ascertainment and control selection. Give the rationale	5-7	(a)"From this cohort, we selected all probands referred for suspected renal hypouricemia, regardless of whether their serum uric acid (SUA) and fractional excretion of UA (FEUA) values met classical diagnostic thresholds." "Relatives of these probands were invited to participate for co-segregation analyses." Additional carriers were identified by screening individuals tested for indications other than suspected renal hypouricemia, including RENAL and NON-RENAL groups. (Methods – Study Participants and Recruitment)

		for the choice of cases and controls *Cross-sectional study*—Give the eligibility criteria, and the sources and methods of selection of participants		
		(*b*) *Cohort study*—For matched studies, give matching criteria and number of exposed and unexposed *Case-control study*—For matched studies, give matching criteria and the number of controls per case		Not applicable (no matched design).
Variables	7	Clearly define all outcomes, exposures, predictors, potential confounders, and effect modifiers.	6–8	Clinical variables included age, sex, SUA, FEUA, plasma and urine creatinine, family history, and disease-related complications. Mild hypouricemia was defined as SUA between 2 and ~3.3 mg/dL with FEUA >10%, while classic hypouricemia was defined as SUA <2 mg/dL with FEUA >10%. Exposure/predictor was pathogenic/likely pathogenic allele count in SLC22A12 or SLC2A9 (0, 1, or 2 alleles). Potential modifiers included urate-modifying therapy, advanced chronic kidney disease, and coexisting genetic variants

		Give diagnostic criteria, if applicable		affecting SUA/FEUA. (Methods – Clinical Characterization; Allele-Dose Analyses)
Data sources/ measurement	8*	For each variable of interest, give sources of data and details of methods of assessment (measurement). Describe comparability of assessment methods if there is more than one group	6–8; Supplementary Methods	Clinical and biochemical data were collected from medical records. Genetic testing was performed by targeted panels or whole-exome sequencing, all including *SLC22A12* and *SLC2A9*; carrier testing in relatives was performed by Sanger sequencing. Assay details and additional urate-handling genes sequenced are provided in Supplementary Table S1. Descriptive statistics and preprocessing are described in the Supplementary Methods.
Bias	9	Describe any efforts to address potential sources of bias	6–8, 14–15; Supplementary Methods	The study explicitly acknowledges referral/ascertainment bias because NephroGalGen is a referral-based genetic testing cohort. To reduce classification bias, variant-negative probands were excluded from the non-carrier group and from allele-count comparisons. Individuals with major urate-interpretation modifiers, including urate-modifying therapy, advanced CKD, *HNF1B*/17q12 deletion, or Gitelman syndrome, were excluded from allele-count group testing. Analyses were displayed with sex-stratified plots and complemented by sensitivity analyses.
Study size	10	Explain how the study size was arrived at	5–6, 9; Figure 1	The study started from the NephroGalGen referral-based genetic testing cohort of approximately 5,400 individuals referred between 2013 and 2025. All probands referred for suspected renal hypouricemia were included. The remaining cohort was screened to identify additional carriers of candidate disease-causing variants. Participant flow and group sizes are summarized in Figure 1.

Quantitative variables	11	Explain how quantitative variables were handled in the analyses. If applicable, describe which groupings were chosen and why	6–8; Supplementary Methods	SUA, FEUA, plasma creatinine, and urine creatinine were analyzed as quantitative variables. SUA was also interpreted using operational categories: classic hypouricemia (SUA <2 mg/dL with FEUA >10%) and mild hypouricemia (SUA 2 to ~3.3 mg/dL with FEUA >10%). Individuals were grouped by pathogenic/likely pathogenic allele count (0, 1, or 2 alleles). The upper boundary of ~3.3 mg/dL was used as a pragmatic, data-informed threshold based on the cohort distribution.
Statistical methods	12	(*a*) Describe all statistical methods, including those used to control for confounding	8; Supplementary Methods	The affected gene (*SLC22A12* vs. *SLC2A9*) was first tested using Mann–Whitney tests with Benjamini–Hochberg correction. As no significant differences were detected, subsequent analyses focused on allele count. Comparisons across allele-count groups used Kruskal–Wallis tests followed by Dunn's post hoc analyses. Individuals with major urate-interpretation modifiers were excluded from allele-count comparisons. Complementary sensitivity analyses assessed the potential contribution of sex and age.
		(*b*) Describe any methods used to examine subgroups and interactions	8; Figure 4; Supplementary Methods	Results were displayed with sex-stratified boxplots to assess visual consistency of the allele-dose pattern. Sex-stratified analyses were interpreted cautiously; the male stratified analysis was considered non-informative because subgroup sizes were very small. Sensitivity analyses also included regression-based assessment of the relative contribution of allele count, age, and sex to SUA. Variant-negative probands were overlaid as a separate exploratory group in the SUA–FEUA scatter plot without genotype assignment.
		(*c*) Explain how missing data were addressed	6–8, 10–11; Supplementary Methods	When Onset or Follow-up values were missing, the available value was used; if both were missing, the variable was recorded as N/A. Missing FEUA availability was explicitly acknowledged as a limitation. Individuals with insufficient or uninterpretable clinical/biochemical information were reported descriptively when appropriate but excluded from allele-count comparisons when major urate-interpretation modifiers were present.

		(*d*) *Cohort study*—If applicable, explain how loss to follow-up was addressed *Case-control study*—If applicable, explain how matching of cases and controls was addressed *Cross-sectional study*—If applicable, describe analytical methods taking account of sampling strategy		Not applicable. This was a retrospective referral-based genetic testing cohort with biochemical values recorded at Onset and Follow-up when available, but no systematic longitudinal follow-up or time-to-event analysis was performed.
		(*e*) Describe any sensitivity analyses	8; Supplementary Methods	Sensitivity analyses included sex-stratified visualization and cautious subgroup assessment, noting that the male stratified analysis was non-informative because of very small subgroup sizes. Regression-based assessment of the relative contribution of allele count, age, and sex to SUA was also performed among individuals with available age at debut. Adult-only analyses were performed to address potential age-related differences in SUA. Individuals with major urate-interpretation modifiers were excluded from allele-count group testing.
**Results**				
Participants	13*	(a) Report numbers of individuals at each stage of	9–12; Figure 1	Among approximately 5,400 individuals in NephroGalGen, 21 probands were referred for suspected renal hypouricemia. Candidate disease-causing variants were detected in 15/21 probands. Segregation analysis in 12 relatives identified

		study—eg numbers potentially eligible, examined for eligibility, confirmed eligible, included in the study, completing follow-up, and analysed		seven monoallelic carriers and five non-carriers. Screening the broader cohort identified 9 renal carriers and 49 non-renal carriers. For allele-count comparisons, SUA was available for 26 individuals with known allele count and FEUA for 17 individuals.
		(b) Give reasons for non-participation at each stage	9–12; Supplementary Table S1	Clinical and biochemical data were unavailable for most additionally identified RENAL and NON-RENAL carriers; therefore, only individuals with interpretable clinical information were reported individually in Table 1 and Supplementary Table S1. Variant-negative probands were excluded from allele-count comparisons. Individuals with major urate-interpretation modifiers were excluded from allele-count group testing.
		(c) Consider use of a flow diagram	Figure 1	Participant flow, cohort structure, and group distribution are shown in Figure 1.
Descriptive data	14*	(a) Give characteristics of study participants (eg demographic, clinical, social) and information on exposures and potential confounders	6–12; Table 1; Supplementary Table S1	Participant characteristics included age, sex, SUA, FEUA, plasma and urine creatinine, family history, disease-related complications, genotype, and relevant modifiers of urate interpretation. These data are summarized in Table 1 and Supplementary Table S1.
		(b) Indicate number of participants with missing data for	10–12; Table 1; Supplementary Table S1	Missing data are shown in Table 1 and Supplementary Table S1. FEUA availability was incomplete and is explicitly acknowledged in the Results and Discussion. For allele-count comparisons, SUA was available for 26 individuals with known allele count and FEUA for 17; paired SUA–FEUA values were

		each variable of interest		available for 17 individuals with known allele count and five variant-negative probands.
		(c) *Cohort study*—Summarise follow-up time (eg, average and total amount)	6-7 Table 1, Table S1	When available, values were recorded at two time points: Onset, defined as first documented hypouricemia, and Follow-up, defined as the most recent available measurement. No systematic longitudinal follow-up time was calculated.
Outcome data	15*	*Cohort study*—Report numbers of outcome events or summary measures over time	10–13; Table 4; Figure 4	Biochemical outcome data are summarized by allele count in Table 4 and Figure 4. SUA differed significantly according to allele count, and FEUA also differed by allele count, although interpretation was limited by incomplete FEUA availability. Clinical outcome data were limited: AKI was documented in one variant-negative proband, and nephrolithiasis was recorded in two monoallelic probands.
		Case-control study—Report numbers in each exposure category, or summary measures of exposure		*Not applicable*
		Cross-sectional study—Report numbers of outcome events or summary measures		*Not applicable*
Main results	16	(*a*) Give unadjusted estimates and, if applicable,	12–13; Figure 4; Table 4	SUA differed significantly according to allele count (Kruskal–Wallis p = 9.96×10^{-4}), with significant pairwise differences between non-carriers and monoallelic carriers, non-carriers and biallelic carriers, and monoallelic and

		confounder-adjusted estimates and their precision (eg, 95% confidence interval). Make clear which confounders were adjusted for and why they were included		biallelic carriers. FEUA also differed by allele count (Kruskal–Wallis p = 0.0060), although FEUA availability was limited. The significant differences between monoallelic carriers and non-carriers supported altered urate handling in individuals carrying a single pathogenic/likely pathogenic allele. Pairwise comparisons involving the biallelic group were interpreted cautiously because this group included only three individuals.
		(*b*) Report category boundaries when continuous variables were categorized	6–8	Mild hypouricemia was defined as SUA between 2 and ~3.3 mg/dL with FEUA >10%; classic hypouricemia was defined as SUA <2 mg/dL with FEUA >10%. Individuals were also grouped according to pathogenic/likely pathogenic allele count: 0, 1, or 2 alleles.
		(*c*) If relevant, consider translating estimates of relative risk into absolute risk for a meaningful time period	14–15	Not applicable. The study did not estimate relative risks or absolute risks over time. Outcome data were limited and retrospectively abstracted; therefore, the cohort was not suitable to estimate absolute complication rates or validate specific SUA cut-offs.
Other analyses	17	Report other analyses done—eg analyses of subgroups and interactions, and sensitivity analyses	8, 12–13; Figure 4; Supplementary Methods	Additional analyses included testing whether the affected gene (*SLC22A12* vs. *SLC2A9*) influenced biochemical values, sex-stratified visualization and cautious subgroup assessment, regression-based assessment of allele count, age and sex, adult-only sensitivity analyses, and exploratory SUA–FEUA scatter plot overlay of variant-negative probands without genotype assignment. The variant-negative overlay was exploratory, did not establish a molecular diagnosis, and did not modify the diagnostic yield.

**Discussion**				
Key results	18	Summarise key results with reference to study objectives	13–15	Among 21 probands with clinical suspicion of renal hypouricemia, pathogenic variants were identified in 15 (71%), including three biallelic and twelve monoallelic cases. Three *SLC22A12* variants (p.Y275X, p.S282L, and p.P268S) were newly associated with renal hypouricemia. Monoallelic carriers differed biochemically from non-carriers, supporting altered urate handling in individuals carrying a single pathogenic/likely pathogenic allele, while comparisons involving the biallelic group were interpreted cautiously because of the small group size. Variant-negative probands with paired SUA–FEUA data fell within the range observed for monoallelic carriers, but this observation was exploratory and did not establish a molecular diagnosis.
Limitations	19	Discuss limitations of the study, taking into account sources of potential bias or imprecision. Discuss both direction and magnitude of any potential bias	15–16	The Discussion states that the biallelic group included only three individuals, limiting the precision of pairwise comparisons involving biallelic carriers. It also states that this is a referral-based genetic testing cohort enriched for suspected inherited kidney disease and more extreme biochemical phenotypes and therefore should not be used to infer population prevalence or absolute complication rates. Additional limitations include modest overall sample size, missing FEUA measurements, incomplete clinical information in some carriers, exclusion of individuals with major urate-interpretation modifiers, limited hard outcomes, lack of systematic non-coding variant assessment, and incomplete exclusion of alternative genetic causes in all variant-negative probands.
Interpretation	20	Give a cautious overall interpretation of results considering objectives, limitations, multiplicity of analyses, results	14-16	The interpretation is cautious: monoallelic pathogenic variants were frequently associated with mild hypouricemia, supporting FEUA assessment and follow-up when low SUA is persistent or clinically suggestive. Variant-negative probands with overlapping biochemical profiles are interpreted as molecularly unresolved, without assignment to the monoallelic group or modification of the diagnostic yield. The authors emphasize that their data support vigilance and monitoring but are not sufficient to estimate complication risk or validate definitive SUA cut-offs.

		from similar studies, and other relevant evidence		
Generalisability	21	Discuss the generalisability (external validity) of the study results	16	The Discussion notes that although the cohort is from Galicia, where allele frequencies may be region-specific, the observed allele-dose relationship is consistent with conserved proximal tubular urate transport biology. Public resources including gnomAD and large-scale biobank/GWAS evidence support relevance beyond Galicia, but external validation in population-based or multicenter cohorts is warranted.
**Other information**				
Funding	22	Give the source of funding and the role of the funders for the present study and, if applicable, for the original study on which the present article is based	2, 17	The funding section lists support from Instituto de Salud Carlos III, RICORS2040, FIS/FEDER, PMP21/00109, the Senefro Foundation, and Xunta de Galicia/GAIN. The manuscript states that none of the funders had any role in study design, data collection, analysis, reporting, or the decision to submit for publication.

*Give information separately for cases and controls in case-control studies and, if applicable, for exposed and unexposed groups in cohort and cross-sectional studies.

Note: An Explanation and Elaboration article discusses each checklist item and gives methodological background and published examples of transparent reporting. The STROBE checklist is best used in conjunction with this article (freely available on the Web sites of PLoS Medicine at http://www.plosmedicine.org/, Annals of Internal Medicine at http://www.annals.org/, and Epidemiology at http://www.epidem.com/). Information on the STROBE Initiative is available at www.strobe-statement.org.
